# Cefazolin versus Cloxacillin or Flucloxacillin for Methicillin-Susceptible *Staphylococcus aureus* bacteremia: A Randomized Clinical Trial

**DOI:** 10.1056/NEJMoa2506905

**Published:** 2026-06-18

**Authors:** Todd C. Lee, Lauren A. Barina, Genevieve Walls, Anna L. Goodman, Dafna Yahav, Matthew P. Cheng, Marc Bonten, Asha C. Bowen, Tom Boyles, Nick Daneman, Miquel B. Ekkelenkamp, Nesrin Ghanem-Zoubi, Marjolein P.M. Hensgens, Nynke G.L. Jager, Achim J. Kaasch, Ilse J.E. Kouijzer, Roger J. Lewis, Thomas Lumley, David C. Lye, Emily G. McDonald, Genevieve McKew, Alistair R.D. McLean, Brendan J. McMullan, Zoe K. McQuilten, Susan C. Morpeth, David L. Paterson, Jason A. Roberts, A/Owen J. Robinson, Hiroki Saito, Matthew Scarborough, Jaap ten Oever, Rebecca M. Turner, Jonas Tverring, Sebastiaan J. van Hal, Steve A. Webb, Lynda M. Whiteway, Cesar A. Arias, Andrew Henderson, George S. Heriot, Tom L. Snelling, Kevin Afra, Shabinah S. Ali, Sharon Amit, Nicholas A. Anagnostou, Sophia Archuleta, Stephen J. Aston, Eugene Athan, Nadav Baharav, Anthony D. Bai, A/Bridget E. Barber, Abinayah Baskaran, Michelle M.A. Berkeley, Emma J. Best, Nilesh R. Bhilave, Max G. Bloomfield, Katherine A. Bond, Jennifer Bostock, Carly L. Botheras, Mark A. Boyd, Sophia M.E. Bradshaw, Elizabeth J. Briggs, A/Philip N. Britton, Anita J. Campbell, Beau Z. Carr, James A. Chancellor, Kevin Chen, Elaine Y. Cheong, Ka Lip Chew, Po Ying Chia, Rispah Chomba, Brian S.W. Chong, Chris J.N. Clews, Jonathan M. Cohen, A/Robert J. Commons, Tim Cutfield, Peter Daley, Diane S. Daniel, Jane Davies, Partha De, Justin T. Denholm, Mia Di Virgilio, Yael Dishon-Benattar, Ravindra Dotel, Erick H. Duan, Nicholas Easom, Noa Eliakim Raz, Michelle M. England, Mark Fahmy, Aidan R. Findlater, Katie L. Flanagan, Ethan Z.J. Foo, Hong Foo, Daniel P. Forster, Michael Fralick, Jaimie L. Frazer, Andrew P. Gador-Whyte, Katherine Garnham, Greg J. German, Niladri Ghosh, Elisabeth H. Gisolf, Stefano G. Giulieri, Susan R. Goulding, Jennifer M. Grant, Daniel Gregson, Kate C. Grimwade, Mordechai Grupper, Stephen D. Guy, A/Amanda Gwee, Victoria G. Hall, Erica J. Hardy, David J. Harris, James Hatcher, Mark R. Hobbs, Natasha E. Holmes, Benjamin P. Howden, Paul J. Huggan, Zoe A. Jennings, Jennie Johnstone, Thomas Juniper, Shirin Kalimuddin, Musaiwale Kamfose, Christopher Kandel, Matthew J. Kelly, Robert A. Kozak, Michelle Kümin, Francois Lamontagne, Jillian S.Y. Lau, Ivor Russel Lee, Ray J. Lin, Daisy Lindsay, Martin J. Llewelyn, Yves Longtin, Sylvain A. Lother, Christopher E. Luey, Derek R. MacFadden, Andrew A. Mahony, Catherine Malden, Isabelle Malhamé, A/Laurens A. Manning, Michael Marks, Leslie J. Martin, Gail V. Matthews, Patrick H. McGann, James H. McMahon, Alexandra Melon, Vidthiya Menon, Dominik Mertz, Michael P. Meyer, James S. Molton, Jocelyn M. Mora, Ed Moran, Leanne M. Mortimer, Satwik Motaganahalli, Matthew P. Muller, Alasdair P.S. Munro, Fionnuala A. Murray, Srinivas Murthy, Vanathi Nagendra, Henco Nel, David W.J. New, Vi Nguyen, Grace Norton, Katherine M. Norton, Clare B. Nourse, Clemency J.S. Nye, Kevin O’Callaghan, Matthew V.N. O’Sullivan, Sean W.X. Ong, Akaninyene A. Otu, Melissa Owen, Jesse Papenburg, Leighanne O Parkes, Mical Paul, Santiago Perez-Patrigeon, Neta Petersiel, Lina Petrella, Sarah L. Pett, David Pham, Chiara J. Piazzese, Sebastien Poulin, Renata Rehak, Elissa Rennert-May, Alexander J. Richards, A/Benjamin A. Rogers, Euna Sahng, Brenda Salada, Oded Scheuerman, Kellie Schneider, Thomas R. Schulz, Kevin L. Schwartz, Peter A. Simos, Harsimran Singh, Fleur S. Sinkeler, Simon Smith, Benjamin J. Smith, Stephanie W. Smith, Ranjani Somayaji, Christine Sommerville, Rima X.J. Song, David C. Sowden, Michael J. Stark, Neil R.H. Stone, Tobias Strunk, A/Archana Sud, Khine P. Swe, Caitlin R. Symons, Yen Ee Tan, Thijs ten Doesschate, Siew Yee Thien, Ashmitha Thomas, Mohamad-Ali Trad, Adrian R. Tramontana, Jennifer L.Y. Tsang, Kimberly Ulett, Jonathan Underwood, Berend J. Van Welzen, Gloria Vazquez-Grande, Diana C. Velasquez Reyes, Janneke D.M. Verberk, Lesley M. Voss, Denise Ward, A/Rachel H Webb, Timothy J. Whitmore, Anat Wieder-Finesod, Heather L. Wilson, Evan W. Wilson, Hei Man Wong, Kate Wong She, Terence Wuerz, Deborah L. Yamamura, Alastair Yeoh, Boris Long Hei Yow, Michael Dymock, Robert K. Mahar, Anna McGlothlin, Julie A. Marsh, Joshua S. Davis, Steven Y.C. Tong, the Staphylococcus aureus Network Adaptive Platform (SNAP) Trial Group

**Affiliations:** 1 Department of Medicine, Division of Infectious Diseases, McGill University, Montreal, Quebec, Canada; 2 Department of Infectious Diseases, The University of Melbourne at the Peter Doherty Institute for Infection and Immunity, 792 Elizabeth Street, Melbourne, Victoria, 3000, Australia; 3 Department of Infectious Diseases, Division of Medicine, Middlemore Hospital, Private Bag 93311, Otahuhu, Auckland, 1640, New Zealand; 4 MRC Clinical Trials Unit, Syndromics, University College London, London, WC1V 6LJ, United Kingdom; 5 Department of Infection, Guy’s & St Thomas’ NHS Foundation Trust and King’s College London, Westminster Bridge Road, London, SE1 7EH, United Kingdom; 6 Microbiology laboratory, Sheba Medical Center, 2 Sheba road, Ramat-Gan, 52621, Israel; 7 Faculty of Medical & Health Sciences, Tel Aviv University, Tel Aviv, Israel; 8 Department of Medicine, Division of Infectious Diseases, McGill University, 1001 Decarie boulevard, Montreal, Quebec, H4A 3J1, Canada; 9 Epidemiology, Julius Center for Health Sciences and Primary Care, University Medical Center Utrecht, Utrecht University, Heidelberglaan 100, Utrecht, 3584 CX, The Netherlands; 10 Ecraid, Archimedeslaan 6, Utrecht, 3584 BA, The Netherlands; 11 Wesfarmers Centre for Vaccines and Infectious Diseases, The Kids Research Institute Australia, Nedlands, 6009, Australia; 12 Department of Infectious Diseases, Perth Children’s Hospital, Nedlands, 6009, Australia; 13 Clinical HIV Research Unit, Faculty of Health Sciences, University of the Witwatersrand, Johannesburg, 2050, South Africa; 14 Medicine, Infectious Diseases, Sunnybrook Health Sciences Centre, University of Toronto, 2075 Bayview Avenue, Toronto, Ontario, M4N 3M5, Canada; 15 Medical Microbiology, Laboratories and Genetics, University Medical Center Utrecht, Heidelberglaan 100, Utrecht, 3584CX, The Netherlands; 16 Infectious Diseases Institute, Rambam Health Care Campus, Haifa, Israel; 17 The Ruth and Bruce Rappaport Faculty of Medicine, Technion, Israel Institute of Technology, Haifa, Israel; 18 Department of Infectious diseases, UMC Utrecht, Utrecht, The Netherlands; 19 Julius Center for Health Sciences and Primary Care, UMC Utrecht, Utrecht, The Netherlands; 20 Pharmacy, Pharmacology & Toxicology, Radboudumc, Nijmegen, The Netherlands; 21 Medical Faculty of the Otto von Guericke University Magdeburg, Institute of Medical Microbiology and Hospital Hygiene, Leipziger Straße 44, Magdeburg, 39120, Germany; 22 Health Campus Immunology, Infection and Inflammation (GCI), Otto von Guericke University Magdeburg, Leipziger Straße 44, Magdeburg, 39120, Germany; 23 Internal Medicine, Infectious Diseases, Radboudumc, Nijmegen, The Netherlands; 24 Berry Consultants, LLC, 3345 Bee Caves Rd #201, Austin, Texas, 78746, United States; 25 Emergency Medicine, David Geffen School of Medicine at UCLA, 1100 Glendon Avenue, Suite 1200, Los Angeles, California, 90024, United States; 26 Statistics, University of Auckland, Auckland, New Zealand; 27 National Centre for Infectious Diseases, Tan Tock Seng Hospital, 16 Jalan Tan Tock Seng, Singapore, 308442, Singapore; 28 Lee Kong Chian School of Medicine, Nanyang Technological University, 11 Mandalay Road, Singapore, Singapore, 308232, Singapore; 29 Department of Medicine, Division of General Internal Medicine, Research Institute of the McGill University Health Centre, 1001 Décarie, Montreal, Quebec, H4A3J1, Canada; 30 Department of Microbiology and Infectious Diseases, Concord Repatriation and General Hospital, NSW Health Pathology, Concord, New South Wales, 2139, Australia; 31 Concord Clinical School, Faculty of Medicine and Health, University of Sydney, Concord, New South Wales, 2139, Australia; 32 Centre for Epidemiology and Biostatistics, Melbourne School of Population and Global Health, University of Melbourne, Melbourne, VIC, Australia; 33 Methods and Implementation Support for Clinical and Health (MISCH) Research Hub, Faculty of Medicine, Dentistry and Health Sciences, University of Melbourne, Melbourne, VIC, Australia; 34 Faculty of Medicine and Health, University of New South Wales, Sydney, NSW, 2052, Australia; 35 Department of Infectious Diseases, Sydney Children’s Hospital, Randwick, Sydney, NSW, 2031, Australia; 36 School of Public Health and Preventive Medicine, Monash University, Melbourne, Australia; 37 Haematology, Alfred Hospital, Melbourne, Australia; 38 Departments of Microbiology and Infectious Diseases, Middlemore hospital, Auckland, New Zealand; 39 Faculty of Medical and Health Sciences, University of Auckland, Auckland, New Zealand; 40 ADVANCE-ID, Saw Swee Hock School of Public Health, National University of Singapore, Singapore, Singapore; 41 Infectious Diseases Translational Research Programme, Yong Loo Lin School of Medicine, National University of Singapore, Singapore, Singapore; 42 University of Queensland Centre for Clinical Research, Faculty of Health Medicine and Behavioural Sciences, The University of Queensland, Herston Rd, Brisbane, Queensland, 4006, Australia; 43 Herston Infectious Diseases Institute, Metro North Health, Butterfield St, Brisbane, Queensland, 4029, Australia; 44 Infectious Diseases, Royal Perth Hospital, Wellington street, Perth, Western Australia, 6000, Australia; 45 Infectious Diseases, Fiona Stanley Hospital, 11 Robin Warren Drive, Murdoch, Western Australia, 6152, Australia; 46 Department of Emergency and Critical Care Medicine, St. Marianna University School of Medicine, Kawasaki, Kanagawa, Japan; 47 Interdepartmental Division of Critical Care Medicine, University of Toronto, Toronto, Canada; 48 Infectious Diseases, MRC, Oxford University Hospitals NHS Foundation Trust, Headington, Oxford, OX3 9DU, UK; 49 Internal Medicine, Radboud University Medical Center, Geert Grooteplein zuid 10, Nijmegen, 6525 GA, The Netherlands; 50 Radboudumc Community for Infectious Diseases, Geert Grooteplein Zuid 10, Nijmegen, 6525 GA, The Netherlands; 51 MRC Clinical Trials Unit, UCL, London, UK; 52 Department of Clinical Sciences Lund/Clinical Sciences Helsingborg, Faculty of Medicine, Infection Medicine, Svartbrödragränden 3, Helsingborg, Skåne, 25187, Sweden; 53 Department of Infectious Diseases, Helsingborg Hospital, Charlott Yhlens gata 10, Helsingborg, Skåne, 25187, Sweden; 54 Department of Infectious Diseases and Microbiology, NSW Health Pathology, Royal Prince Alfred Hospital, Sydney, NSW, 2050, Australia; 55 Central Clinical School, University of Sydney, Sydney, NSW, 2050, Australia; 56 Australian and New Zealand Intensive Care Research Centre, Monash University, St Kilda Rd, Melbourne, Victoria, 3004, Australia; 57 St John of God Health Care, Wellington St, Perth, Western Australia, 6000, Australia; 58 Internal Medicine, Infectious Diseases, Houston Methodist and Weill Cornell Medical College, 6560 Fannin St, Sucrlock Tower, Suite 1512, Houston, Texas, 77030, United States; 59 Department of Medicine, Weill Cornell Medical College, 1300 York Avenue, New York, 10021, United States; 60 Infection Management Services, Princess Alexandra Hospital Brisbane, Australia; 61 Department of Infectious Diseases, Melbourne Medical School, University of Melbourne, Grattan St, Parkville, VIC, 3010, Australia; 62 School of Public Health, University of Sydney, Camperdown, NSW, Australia; 63 Department of Infectious Diseases, Children’s Hospital at Westmead, Westmead, NSW, Australia; 64 Department of Medicine, Division of Infectious Diseases, Fraser Health, Surrey, British Columbia, Canada; 65 MRC Clinical Trials Unit, Institute of Clinical Trials & Methodology, UCL, 90 High Holborn, London, WC1V 6LJ, UK; 66 The Faculty of Medical and Health Sciences, Tel-Aviv University, Te-Aviv, Israel; 67 Microbiology and Infectious Diseases, Medicine, Cardiac and Critical Care, Southern Adelaide Local Health Network, Flinders Drive, Bedford Park, South Australia, 5042, Australia; 68 Department of Medicine, Division of Infectious Diseases, National University Hospital, Singapore, Singapore; 69 Yong Loo Lin School of Medicine, National University of Singapore, Singapore; 70 Tropical and Infectious Diseases Unit, University Hospitals Liverpool Group, Mount Vernon Street, Liverpool, L7 8YE, United Kingdom; 71 Pharmacology and Therapeutics, Institute of Systems, Molecular and Integrative Biology, University of Liverpool, William Henry Duncan Building, 6 West Derby Street, Liverpool, L7 8TX, United Kingdom; 72 Infectious Disease, Medicine, Centre for Innovation in Infectious Disease and Immunology Research, Deakin University, Bellarine St, Geelong, Victoria, 3220, Australia; 73 Faculty of Medicine, Tel Aviv University, Te-Aviv, Israel; 74 Department of Medicine, Division of Infectious Diseases, Queen’s University, Kingston, Ontario, Canada; 75 Infectious Diseases, Royal Brisbane & Women’s Hospital, Brisbane, Queensland, 4006, Australia; 76 QIMR Berghofer Medical Research Institute, Brisbane, Queensland, 4006, Australia; 77 Medical Research Council Clinical Trials Unit at University College London, Institute of Clinical Trials and Methodology, London, United Kingdom; 78 Infection Research, Infection Division - Medicine Board, University College London Hospital Trust Foundation, 250 Euston Road, London, London, NW1 2PG, United Kingdom; 79 Department of Pediatric Infectious Diseases, Starship Children’s Health, Park Road, Auckland, New Zealand; 80 Department of Pedaitrics; Child and Youth Health, Faculty of Medical and Health Sciences, The University of Auckland, Park Road, Auckland, New Zealand; 81 Goulburn Valley Hospital, Graeme Street, Shepparton, Victoria, 3630, Australia; 82 Infection Services, Te Whatu Ora - Capital, Coast, Hutt Valley & Wairarapa, Wellington, New Zealand; 83 Departments of Microbiology and Infectious Disease, Royal Melbourne Hospital, 300 Grattan St, Parkville, Victoria, 3052, Australia; 84 Victorian Infectious Diseases Reference Laboratory, The University of Melbourne at the Peter Doherty Institute for Infection and Immunity, 792 Elizabeth St, Melbourne, Victoria, 3000, Australia; 85 Care Policy and Evaluation Centre, The London School of Economics and Political Science, London, WC2A 2AE, UK; 86 Infectious Disease, Barwon Health, Geelong, 3220, Australia; 87 School of Medicine, Faculty of Health and Medical Sciences, University of Adelaide, Adelaide, South Australia, Australia; 88 Infectious Disease Unit, Division of Medicine, Northern Adelaide Local Health Network, Haydown Road, Elizabeth Vale, Adelaide, 5112, Australia; 89 Medical Research Council Clinical Trials Unit at University College London, University College London Institute of Clinical Trials and Methodology, 90 High Holborn, London, WC1E 6BT, United Kingdom; 90 Adult Infectious Diseases, Te Toka Tumai Auckland City Hospital, Auckland, New Zealand; 91 Department of Infectious Diseases and Microbiology, The Children’s Hospital at Westmead, Locked Bag 4001, Westmead, NSW, 2145, Australia; 92 Sydney Medical School, Faculty of Medicine and Health, University of Sydney, Sydney, NSW, 2000, Australia; 93 Infectious Diseases Department, Perth Children’s Hospital, 15 Hospital Ave, Nedlands, Western Australia, 6009, Australia; 94 Wesfarmers Centre of Vaccines and Infectious Diseases, The Kids Research Institute Australia, 15 Hospital Ave, Nedlands, Western Australia, 6009, Australia; 95 Infectious Diseases, Medicine, Eastern Health, 8 Arnold St, Box Hill, Victoria, 3128, Australia; 96 Infectious Diseases, Tauranga Hospital, Tauranga, Bay of Plenty, 3112, New Zealand; 97 Sydney Medical School, The University of Sydney, Sydney, New South Wales, Australia; 98 Department of Laboratory Medicine, Division of Microbiology, National University Hospital, Singapore, Singapore; 99 Department of Infectious Diseases, National Centre for Infectious Diseases, Singapore, Singapore; 100 Department of Infectious Diseases, Tan Tock Seng Hospital, Singapore; 101 Clinical Microbiology and Infectious Diseases (CMID), Faculty of Health Sciences, University of the Witwatersrand, 29 Princess of Wales Terrace, Parktown, Johannesburg, 2193, South Africa; 102 Clinical microbiology, Helen Joseph hospital, National Health Laboratory Services (NHLS), 1 Perth Road Auckland Park, Rossmore, Johannesburg, 2092, South Africa; 103 Department of Infectious Diseases, John Hunter Hospital, Newcastle, NSW, 2301, Australia; 104 Dept Paediatric Immunology & Infectious Diseases, Evelina London Children’s Hospital, Guys & St Thomas’ NHS Foundation Trust, London, UK; 105 Dept Women & Children’s Health, King’s College London, London, UK; 106 General and Subspecialty Medicine, Grampians Health Ballarat, Ballarat, Victoria, 3350, Australia; 107 Global and Tropical Health Division, Menzies School of Health Research and Charles Darwin University, Darwin, Northern Territory, 810, Australia; 108 Infectious Diseases, Memorial University of Newfoundland, St John’s, Newfoundland, A1B 3V6, Canada; 109 Department of Microbiology and Immunology, Microbiological Diagnostic Unit Public Health Laboratory, Peter Doherty Institute for Infection & Immunity, The University of Melbourne, 792 Elizabeth Street, Melbourne, Victoria, 3000, Australia; 110 Global and Tropical Health, Menzies School of Health Research, Charles Darwin University, Rocklands Drive, Darwin, NT, 0810, Australia; 111 Infectious Diseases, Medicine, Royal Darwin Hospital, Rocklands Drive, Darwin, NT, 0810, Australia; 112 Microbiology, Department of Laboratory Medicine, Tan Tock Seng Hospital, Singapore, 308433, Singapore; 113 Lee Kong Chian School of Medicine, Nanyang Technological University, Singapore, 639798, Singapore; 114 Victorian Infectious Diseases Service, Royal Melbourne Hospital, Grattan Street, Parkville, VIC, 3050, Australia; 115 The University of Melbourne at the Peter Doherty Institute for Infection and Immunity, 792 Elizabeth Street, Parkville, VIC, 3050, Australia; 116 Infectious Diseases Institute, Rambam Health Care Campus, HaAliya HaShniya St 8, Haifa, 3109601, Israel; 117 The Cheryl Spencer Department of Nursing, University of Haifa, Haifa, Israel; 118 Infectious Diseases, Blacktown Hospital, Sydney, Australia; 119 Department of Medicine, Division of Critical Care, McMaster University, St Catherines, Ontario, Canada; 120 Infection Research Group, Department of Infection, Hull University Teaching Hospitals NHS Trust, Anlaby Road, Hull, HU3 2JZ, UK; 121 Hull York Medical School, University of Hull, Hull, HU6 7RX, UK; 122 Internal Medicine E, Unit of Infectious Diseases, Rabin Medical Center; Beilinson Campus, Petach-Tikvah, 49100, Israel; 123 Department of Medicine, Division of Infectious Diseases, McMaster University, Hamilton, Ontario, Canada; 124 School of Medicine, University of Tasmania, Liverpool Street, Hamilton, Tasmania, 7000, Australia; 125 School of Health and Biomedical Sciences, RMIT University, Plenty Road, Melbourne, Victoria, 3082, Australia; 126 Department of Microbiology and Infectious Diseases, NSW Health Pathology - Liverpool, Sydney, Australia; 127 Monash University, Wellington Rd, Clayton, Victoria, 3800, Australia; 128 Division of General Internal Medicine Sinai Health, University of Toronto, Toronto, Ontario, Canada; 129 Department of infectious Diseases, Division of Medicine, Launceston General Hospital, 270 Charles Street, Launceston, Tasmania, 7250, Australia; 130 Department of Infectious Diseases and Immunology, Austin Health, 145 Studley Road, Heidelberg, Victoria, 3084, Australia; 131 Infectious Diseases, Bendigo Health, Bendigo, Victoria, 3550, Australia; 132 Department of Infectious Diseases, Gold Coast University Hospital, Southport, Queensland, 4215, Australia; 133 Department of Laboratory Medicine, Division of Microbiology, St. Joseph’s Health Centre (Unity Health Toronto), Toronto, Ontario, Canada; 134 Department of Infectious Diseases, Wollongong Public Hospital, Wollongong, New South Wales, 2500, Australia; 135 Infectieziekten, Interne Geneeskunde, Rijnstate ziekenhuis, Wagnerlaan 55, Arnhem, 6815AD, Netherlands; 136 Department of Microbiology and Immunology, The University of Melbourne, Melbourne, Australia; 137 Victorian Infectious Diseases Department, The Royal Melbourne Hospital, Melbourne, Australia; 138 Internal Medicine, Infectious Diseases, University of British Columbia, Vancouver, BC, V5Z 1M9, Canada; 139 Medicine, Infectious Diseases, University of Calgary, Calgary, Alberta, T2K 1Y1, Canada; 140 Pathology and Laboratory Medicine, Medical Microbiology, University of Calgary, Calgary, Alberta, T2Y 1Y1, Canada; 141 Eastern Health Clinical School, Monash University, Victoria, Australia; 142 Antimicrobials Group, Murdoch Children’s Research Institute, Melbourne, Australia; 143 Paediatrics, University of Melbourne, Australia, Melbourne, Victoria, 3052, Australia; 144 Infectious Diseases, Peter MacCallum Cancer Centre, Melbourne, Victoria, 3000, Australia; 145 Medicine, Infectious Diseases, University of Toronto, Toronto, Ontario, Canada; 146 Medicine, Infectious Disease, Women and Infants Hospital, 101 Dudley St, Providence, RI, 02905, USA; 147 Department of Medicine, Division of Infectious Diseases, University of British Columbia, Vancouver, British Columbia, Canada; 148 Department of Microbiology and Virology, Great Ormond Street Hospital for Children, London, United Kingdom; 149 Infection, Inflammation, and Immunity Department, University College London, London, United Kingdom; 150 Microbiological Diagnostic Unit Public Health Laboratory, Department of Microbiology and Immunology, The University of Melbourne at the Peter Doherty Institute for Infection and Immunity, 792 Elizabeth Street, Melbourne, Victoria, 3000, Australia; 151 Centre for Pathogen Genomics, The University of Melbourne, Melbourne, Victoria, 3010, Australia; 152 Department of Infectious Disease, Waikato Hospital, Pembroke Street, Hamilton, Waikato, 3204, New Zealand; 153 Infectious Diseases, Medicine, Nepean Hospital, University of Sydney, Derby Street, Kingswood, NSW, 2747, Australia; 154 Department of Medicine, Division of Infectious Diseases, University of Toronto, Toronto, Ontario, Canada; 155 Department of Infection, Guy’s and St Thomas’ Hospitals NHS Foundation Trust, London, UK; 156 Department of Infectious Diseases, Singapore General Hospital, 20 College Road, Singapore, 169608, Singapore; 157 Research, Oxford University NHS Foundation Trust, Oxford, United Kingdom; 158 Department of Medicine, Division of Infectious Diseases, Michael Garron Hospital, Toronto, Ontario, Canada; 159 Department of Medicine, Hutt Hospital, 638 High Street, Lower Hutt, 5010, New Zealand; 160 Biological Sciences, Sunnybrook Research Institute, Sunnybrook Health Sciences Centre, Toronto, Ontario, Canada; 161 Experimental Medicine, Nuffield Department of Medicine, University of Oxford, Level 5, Room 5800, John Radcliffe Hospital, Oxford, OX3 9DU, UK; 162 Department of Medicine, Division of Internal Medicine, CIUSSSE-CHUS, Sherbrooke, Quebec, Canada; 163 Department of Infectious Diseases, Monash Health, 246 Clayton Road, Clayton, VIC, 3168, Australia; 164 Singapore Infectious Disease Clinical Research Network, National Centre for Infectious Diseases, Singapore, Singapore; 165 Singapore Infectious Disease Clinical Research Network, Communicable Diseases Agency, Singapore, Singapore; 166 Department of Infectious Diseases, Division of Medicine, Woodlands Health, 17 Woodlands Drive 17, Singapore, 737628, Singapore; 167 Global Health and infection, Brighton and Sussex Medical School, Biology Road, Falmer, East Sussex, BN1 9PS, United Kingdom; 168 Infectious Diseases, Medicine, University Hospitals Sussex NHS Foundation Trust, Eastern Road, Brighton, East Sussex, BN2 5BE, United Kingdom; 169 Division of Infectious Diseases, Jewish General Hospital, Montreal, Quebec, Canada; 170 Department of Internal Medicine, Divisions of Infectious Diseases and Critical Care, University of Manitoba, Winnipeg, Manitoba, Canada; 171 Division of Clinical Epidemiology, Ottawa Hospital Research Institute, Ottawa, Ontario, Canada; 172 Infectious Diseases and Immunology, Austin Health, Heidelberg, Victoria, 3084, Australia; 173 Infectious Diseases Unit, Flinders Medical Centre, Adelaide, Australia; 174 Department of Medicine, Division of General Internal Medicine, Centre for Outcomes Research and Evaluation, McGill University Health Centre, Montreal, Quebec, Canada; 175 Medical School, University of Western Australia, Crawley, Western Australia, 6009, Australia; 176 Clinical Research Department, Faculty of Infectious and Tropical Diseases, London School of Hygiene & Tropical Medicine, London, United Kingdom; 177 Hospital for Tropical Diseases, University College London Hospital, London, United Kingdom; 178 Department of Medicine, Division of General Internal Medicine, McMaster University, Montreal, Quebec, Canada; 179 Infectious Disease Department, St Vincent’s Hospital, Victoria Street, Sydney, NSW, 2010, Australia; 180 Therapeutics and Vaccine Research Program, Kirby Institute, University of New South Wales, Sydney, NSW, 2052, Australia; 181 Infectious diseases, Alfred Hospital, Melbourne, Victoria, 3004, Australia; 182 Infectious diseases, School of Translational medicine, Monash University, Melbourne, Victoria, 3004, Australia; 183 Infectious Diseases, Medicine, Sunshine Coast University Hospital, Doherty Street, Birtinya, QLD, 4575, Australia; 184 Nepean Clinical School, Faculty of Medicine, University of Sydney, NSW, 2006, Australia; 185 Department of Medicine, Division of Infectious Diseases, McMaster University, 1280 Main St W, Hamilton, ON, L8S 4L8, Canada; 186 KizFirst, Neonatology, Middlemore Hospital, 100 Hospital Road, Otahuhu, Auckland, 1025, New Zealand; 187 Infectious Diseases, Western Health, Gordon St, Footscray, Victoria, 3011, Australia; 188 Doherty Institute, University of Melbourne, Melbourne, VIC, Australia; 189 Department of Infectious Disease, Division of Medicine, North Bristol NHS Trust, Southmead Road, Bristol, BS10 5NB, United Kingdom; 190 Faculty of Medicine, Pathology and Laboratory Medicine, University of Ottawa, Ottawa, Ontario, Canada; 191 Clinical and Experimental Sciences, Faculty of Medicine, University of Southampton, University Road, Southampton, Hampshire, SO171BJ, United Kingdom; 192 NIHR Southampton Clinical Research Facility and Biomedical Research Centre, University Hospital Southampton NHS Foundation Trust, Tremona road, Southampton, Hampshire, SO166YD, United Kingdom; 193 Pediatrics, University of British Columbia, Vancouver, Canada; 194 Infectious Diseases Department, Armadale Hospital, 3056 Albany Highway, Mt Nasura, Western Australia, Australia; 195 School of Medicine and Public Health, University of Newcastle, Callaghan, Australia; 196 Infection Research Program, Hunter Medical Research Institute, Australia; 197 Paediatric Infectious Disease, Medicine, Queensland Childrens Hospital, Stanley, Brisbane, Queensland, 4101, Australia; 198 School of Medicine, Medicine, University of Queensland, St Lucia, Brisbane, Queensland, 4067, Australia; 199 Infectious Disease, University hospital of Wales, Cardiff, United Kingdom; 200 Department of Infectious Diseases, Redcliffe Hospital, Redcliffe, Queensland, 4020, Australia; 201 Centre for Infectious Diseases and Microbiology, Westmead Hospital, Hawkesbury Road, Westmead, New South Wales, 2145, Australia; 202 Sydney Infectious Diseases Institute, Faculty of Medicine and Health, University of Sydney, Camperdown, New South Wales, 2006, Australia; 203 Department of Infectious Diseases, University of Melbourne, at the Peter Doherty Institute for Infection and Immunity, Melbourne, Australia; 204 Institute of Health Policy, Management and Evaluation, University of Toronto, Toronto, Ontario, Canada; 205 Pediatrics, Pediatric Infectious Diseases, Montreal Children’s Hospital, McGill University Health Centre, 1001 Blvd Décarie, Montreal, Quebec, H4A 0B1, Canada; 206 The Ruth and Bruce Rappaport Faculty of Medicine, Technion – Israel Institute of Technology, Efron St 1, Haifa, 3525433, Israel; 207 Department of Medicine, Infectious Diseases, Queen’s University, Kingston, Ontario, Canada; 208 Department of Medicine, Division of Infectious Diseases, Research Institute of the McGill University Health Centre, Montreal, Quebec, Canada; 209 MRC CTU at UCL, Institute of Clinical Trials and Methodology, 90 High Holborn, London, WC1V6LJ, United Kingdom; 210 Sydney Infectious Diseases Institute, The University of Sydney, Westmead, NSW, 2145; 211 CISSS des Laurentides, Hôpital de St-Jérôme, Unité de recherche clinique du CISSS des Laurentides, St-Jérôme, Quebec, Canada; 212 Department of Medicine, University of Calgary, Calgary, Alberta, Canada; 213 Departmen of Medicine, Division of Infectious Diseases, University of Calgary, Calgary, Alberta, Canada; 214 Hull York Medical School, Hull, HU6 7RX, United Kingdom; 215 School of Clinical Sciences at Monash Health, Monash University, 246 Clayton Road, Clayton, Victoria, 3168, Australia; 216 Monash Infectious Diseases, Monash Health, 246 Clayton Road, Clayton, Victoria, 3168, Australia; 217 Division of Infectious Diseases, Department of Medicine, National University Hospital, Singapore, Singapore; 218 Pediatrics B, Pediatric Division, Schneider children medical center Israel, 14 kaplan, ramat gan, israel; 219 Faculty of medical &health sciences, Tel-Aviv university, Tel Aviv, israel; 220 Victorian Infectious Diseases Service (VIDS), Medicine, Peter Doherty Institute for Infection and Immunity (PDI), Gratton Street, Parkville, Victoria, 3050, Australia; 221 Medicine, Infectious Diseases, St. Joseph’s Health Centre - Unity Health Toronto, 30 The Queensway, Toronto, Ontario, M6R 1B5, Canada; 222 Dalla Lana School of Public Health, University of Toronto, 155 College St, Toronto, Ontario, M5T 3M7, Canada; 223 Department of Pharmacy, Pharmacology and Toxicology, Radboud university medical center, Geert Grooteplein Zuid 10, Nijmegen, 6525 GA, The Netherlands; 224 Department of Medicine, Cairns Hospital, Cairns, Queensland, Australia; 225 Department of Medicine, Division of Infectious Diseases, University of Alberta, Edmonton, Alberta, Canada; 226 Department of Medicine, Cumming School of Medicine, University of Calgary, Calgary, Alberta, Canada; 227 Infectious Diseases, Doherty Institute, The University of Melbourne, Melbourne, Victoria, Australia; 228 The Robinson Research Institute, The University of Adelaide, Adelaide, South Australia, 5005, Australia; 229 Department of Neonatal Medicine, The Women’s and Children’s Hospital, 72 King William Road, Adelaide, South Australia, 5006, Australia; 230 Division of Infection, University College London Hospitals, London, United Kingdom; 231 Neonatal Directorate, Child and Adolescent Health Service, Perth, Western Australia, 6009, Australia; 232 Wesfarmers Centre for Vaccines and Infectious Diseases, The Kids Research Institute Australia, 15 Hospital Ave, Nedlands, WA, 6009, Australia; 233 Microbiology, Singapore General Hospital, Singapore, Singapore; 234 Department of internal medicine, Divis, Jeroen Bosch Hospital, Henri Dunantstreet1, s-Hertogenbosch, Brabant, 5223 GZ, The Netherlands; 235 Research program Infectious Diseases, Julius Center for Health Sciences and Primary Care, University Medical Center Utrecht, Heidelberglaan 100, Utrecht, Utrecht, 3584 CG, The Netherlands; 236 Department of Infectious Diseases, Division of Medicine, Singapore General Hospital, 1 Hospital Drive, Bukit Merah, Singapore, Singapore, 169608, Singapore; 237 Niagara Health Knowledge Institute, Niagara Health, St Catharines, Ontario, Canada; 238 General Medicine, Gold Coast Hospital and Health Service, 1 Hospital Blvd, Southport, QLD, 4215, Australia; 239 Department of Infectious Diseases, Cardiff and Vale University Health Board, Cardiff, Wales, UK; 240 Division of Infection and Immunity, Cardiff University, Cardiff, Wales, UK; 241 Department of Infectious Diseases, University Medical Centre Utrecht, Utrecht, The Netherlands; 242 Department of Medicine, Section of Critical Care Medicine, University of Manitoba, Winnipeg, Manitoba, Canada; 243 Julius Center for Health Sciences and Primary Care, University Medical Center Utrecht, Utrecht, The Netherlands; 244 European Clinical Research Alliance on Infectious Diseases (Ecraid), Utrecht, The Netherlands; 245 Paediatric Infectious Diseases, Starship Children’s Hospital, Auckland, 1142, New Zealand; 246 MRC Clinical Trials Unit at UCL, Institute of Clinical Trials and Methodology, London, United Kingdom; 247 Department of Paediatrics: Child and Youth Health, Faculty of Medical and Health Sciences, University of Auckland, Park Road, Grafton, Auckland, New Zealand; 248 Kidz First Children’s Hospital, Middlemore Hospital, Health New Zealand: Counties Manukau, Hospital Road, Auckland, New Zealand; 249 Department of Respiratory Medicine, Royal Perth Hospital, Perth, Western Australia, 6000, Australia; 250 Faculty of medicine, Tel-Aviv university, Tel Aviv, Israel; 251 Infectious Diseases and Clinical Microbiology, Division of Medicine, Canberra Hospital, Yamba Drive, Canberra, Australian Capital Territory, 2605, Australia; 252 Department of Infectious Diseases, Division of Medicine, Singapore General Hospital, Singapore, Singapore; 253 Grants, Aotearoa Clinical Trials, Auckland, New Zealand; 254 Department of Medicine, Section of Infectious Diseases, University of Manitoba, Winnipeg, Manitoba, Canada; 255 Department of Pathology and Molecular Medicine, McMaster University, Hamilton, Ontario, Canada; 256 Wesfarmers Centre of Vaccines and Infectious Diseases, The Kids Research Institute Australia, 15 Hospital Avenue, Perth, Western Australia, 6009, Australia; 257 School of Population and Global Health, The University of Western Australia, 35 Stirling Highway, Perth, Western Australia, 6009, Australia; 258 Melbourne School of Population and Global Health, University of Melbourne, Parkville, Victoria, Australia; 259 Clinical Epidemiology and Biostatistics Unit, Murdoch Children’s Research Institute, Parkville, Victoria, Australia; 260 Statistics, Berry Consultants, LLC, Austin, Texas, 78746, USA; 261 Wesfarmers Centre of Vaccines and Infectious Diseases, The Kids Research Institute, 15 Hospital Avenue, Nedlands, Western Australia, 6009, Australia; 262 School of Medicine and Public Health, The University of Newcastle, Newcastle, NSW, 2301, Australia; 263 Victorian Infectious Diseases Service, The Royal Melbourne Hospital, at the Peter Doherty Institute for Infection and Immunity, Melbourne, VIC, 3000, Australia

## Abstract

**Background:**

Whether cefazolin or an antistaphylococcal penicillin should be preferred for treating methicillin-susceptible *Staphylococcus aureus* (MSSA) bacteremia is unclear.

**Methods:**

In an ongoing, international, Bayesian adaptive platform trial, we conducted an open-label, randomized comparison of cefazolin versus an antistaphylococcal penicillin (flucloxacillin or cloxacillin) in adult patients with penicillin-resistant, methicillin-susceptible *S. aureus* bacteremia. The primary outcome was 90-day all-cause mortality, evaluated using a hierarchical Bayesian logistic regression model. We report the posterior probability of non-inferiority (pre-specified as an adjusted odds ratio [aOR] < 1.2, approximating < 2.5% absolute difference if mortality in the (flu)cloxacillin arm was 15%) and of superiority (corresponding to an aOR <1.0). Secondary safety outcomes included the development of acute kidney injury within 14 days.

**Results:**

This trial domain was conducted between February 17, 2022, and August 7, 2024, at which time non-inferiority was met. Death at 90 days amongst evaluable adults was 15.0% (97/645) for cefazolin and 17.0% (109/642) for (flu)cloxacillin (aOR 0.81; 95% Credible Interval [CrI], 0.59–1.12; probability of non-inferiority 99.2% and superiority 89.8%). Acute kidney injury occurred in 92/660 (13.9%) patients in the cefazolin arm versus 127/648 (19.6%) in the (flu)cloxacillin arm (aOR 0.67, 95%CrI 0.50–0.89; probability of superiority 99.7%).

**Conclusion:**

In patients with MSSA bacteremia, cefazolin had a high probability of non-inferiority to (flu)cloxacillin for 90-day mortality, with less acute kidney injury. (Funded by National Health and Medical Research Council and others; Clinicaltrials.gov NCT05137119).

*S. aureus* bacteremia is a leading cause of bacterial-related mortality worldwide^1^ with more than 1 in 4 afflicted patients dying within 90 days.^2^ While there are accepted best practices in the management of *S. aureus* bacteremia,^3^ the preferred antibiotic for treating methicillin-susceptible *S. aureus* (MSSA) is unclear.^4^ Expert opinion from endocarditis guidelines^5,6^ favors the antistaphylococcal penicillins (e.g., nafcillin, oxacillin, cloxacillin, or flucloxacillin) over cefazolin^5^ due to theoretical concerns about the “cefazolin inoculum effect”, an *in vitro* destruction of cefazolin by beta-lactamases which may be relevant in high burden infections.^7,8^ However, meta-analyses of observational studies suggest that cefazolin could be superior in terms of 30-day mortality (odds ratio 0.73; 95%CI 0.62-0.85)^9^ with a more favorable adverse event profile when compared to antistaphylococcal penicillins. Given observational analyses may be subject to multiple biases,^10^ a randomized clinical trial is the most reliable way to inform patient management.

We launched the *S. aureus* Network Adaptive Platform (SNAP) trial in 2022 to answer multiple questions related to the treatment of patients with *S. aureus* bacteremia.^11^ Within this Bayesian adaptive platform trial, we conducted a pragmatic, open-label, randomized comparison of cefazolin to an antistaphylococcal penicillin (cloxacillin or flucloxacillin depending on the country [hereafter (flu)cloxacillin]) for the treatment of MSSA bacteremia.

## Methods

### Study Design and Setting

SNAP contains a series of investigator-initiated, international, multicenter studies.^11^ The platform features a master protocol and integrated statistical framework^12^ with domain specific appendices containing nested questions. Healthcare consumers were involved in trial design through focus groups and representation on steering committees. Each domain^11^ refers to a management question with at least two interventions being compared (e.g., backbone antibiotics, adjunctive clindamycin therapy^13^, or early oral switch^14^; see Supplementary Appendix). The backbone domain is further divided into silos based on *S. aureus* antibiotic susceptibility: penicillin-susceptible (PSSA); methicillin-susceptible, penicillin-resistant (MSSA); and methicillin-resistant (MRSA).

Herein we report the open-label, parallel group, non-inferiority, randomized comparison of patients in the MSSA silo of the antibiotic backbone domain of SNAP. Participants in this domain and silo were recruited between February 17, 2022, and June 21, 2024, at 91 sites in 8 countries (Australia, Canada, Israel, New Zealand, the Netherlands, Singapore, South Africa, and the United Kingdom; see Supplementary Appendix) with the last participant reaching 90-days of follow-up on September 21, 2024. Ethics and regulatory approval were obtained at each participating center. Written or oral informed consent, in accordance with regional regulations, was obtained from all patients and/or their surrogates. The study was designed by the investigators via international working groups. Data was analyzed by a dedicated analytic team of investigators. Tong, Norton, Mahar, McGlothlin, and Dymock vouch for the data and analysis. The study was supported by funding organizations and academic sponsors in multiple countries (Supplementary Appendix) who had no role in the design, analysis, or reporting of the results. The master protocol,^11^ statistical methods,^12^ domain specific appendix, and statistical analysis plan are available in the Supplementary Appendix and the protocol at nejm.org. SNAP is registered on clinicaltrials.gov (NCT05137119).

### Participants

We enrolled hospitalized patients with *S. aureus* bacteremia to the platform within 72 hours of their index blood culture collection. This report describes adults (≥18 years). The pedia tric s tudy is ongoing. Platform and domain eligibility criteria are detailed in the Supplementary Appendix. Notable platform exclusions included enrollment beyond 72 hours from collection of the first positive blood culture, polymicrobial bacteremia, or patients not for active treatment. Key domain specific exclusions included an active history of penicillin or cefazolin allergy; current receipt of maintenance dialysis; and ongoing treatment with other systemic antibacterial agents with *S. aureus* activity. Additionally, because the cefazolin inoculum effect is dependent on the production of penicillinase, we only included penicillin-resistant MSSA (see *Laboratory Methods*) to avoid bias towards non-inferiority of cefazolin.

### Randomization and Allocation

All participants were randomized 1:1 within the MSSA silo at platform entry by simple randomization without stratification using a web-based interactive randomization system (Spiral Software, New Zealand). As soon as participants were confirmed to be eligible for the MSSA silo of the backbone domain, their randomized allocation to cefazolin or (flu)cloxacillin was revealed.

### Interventions

Recommended dosing was: 2g intravenously (IV) q8h for cefazolin (q6h for critical illness or endocarditis); 2g IV q6h for flucloxacillin (q4h for critical illness or endocarditis), and 2g IV q4h for cloxacillin, with dose adjustments for renal function (Supplementary Appendix). Other than potential treatment assignment in the adjunctive antibiotic domain,^13^ post-enrollment administration of additional antibiotics was discouraged.

Infectious diseases consultation was advised. The protocol recommended allocated antibiotics be continued for the entire intravenous treatment duration, with a minimum antibiotic duration of 14 days for uncomplicated bacteremia and 28–42 days for complicated bacteremia.^15^ Subsequent randomization to the oral switch domain^14^ was permitted at 7 and 14 days, respectively for eligible participants. Apart from the antistaphylococcal antibiotics used, clinical management was left to the treating clinician’s discretion.

### Outcomes

The primary outcome for the platform and domain was all-cause mortality at 90 days after platform entry. Secondary platform outcomes included: all-cause mortality at 14, 28, and 42 days; microbiologic treatment failure and diagnosis of new foci of infection between days 15 and 90; and *Clostridioides difficile* infection within 90 days. Secondary domain specific outcomes (further defined in Supplementary Appendix) included acute kidney injury (AKI), defined as an absolute creatinine increase ≥26.5 μmol/L (0.3mg/dL) within 5 days or a relative increase ≥50% from baseline within the first 14 days, initiation of renal replacement therapy, and hepatotoxicity.

### Adverse Events

In addition to the pre-specified safety outcomes, site investigators were asked to conform to regional requirements and, at a minimum, record serious adverse reactions defined as all serious adverse events that were thought to be possibly, probably, or definitely related to study drugs or study procedures.

### Laboratory Methods

Methicillin and penicillin susceptibility were determined locally using automated testing methods and/or phenotypic disc diffusion^16^ and/or molecular testing for the *mecA* and *blaZ* genes (Supplementary Appendix).

### Statistical Methods

#### Study Populations

Populations were defined based on the principle of intention-to-treat (ITT) for each estimand corresponding to the revealed randomized treatment assignment, regardless of receipt. The primary analysis was also performed on a protocol-adherent population (Supplementary Appendix).

#### Analyses

The scheduled and final analyses were performed by an independent unblinded analytic team which was separate from the trial team. For this analysis, we used a minimally-informative prior for all model parameters. The analyses are described in the statistical appendix included with the Supplementary Appendix.

**Figure 1:**
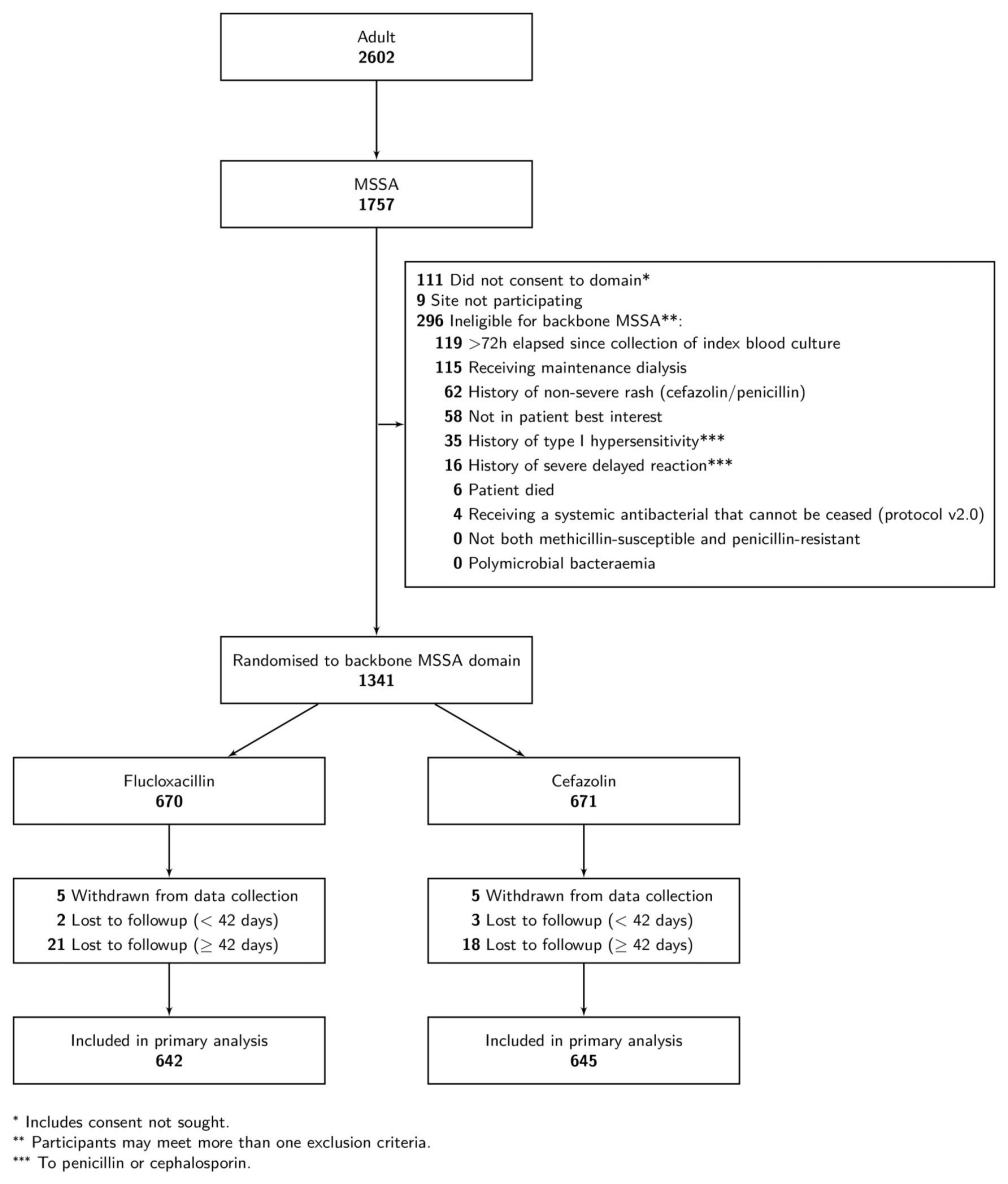
Enrolment, randomization, and follow-up. Notes: MSSA denotes methicillin-susceptible Staphyloccocus aureus.

The adjusted odds ratio (aOR) for the primary outcome was calculated using a hierarchical Bayesian logistic regression model. This model incorporated data from all platform participants to adjust for age, country, temporal epoch (contiguous 26-week periods from the start of the domain), and eligibility and treatment assignment in other platform domains. Details of the model fitting and diagnostics are provided in the Supplementary Appendix. The treatment effect pertaining to cefazolin versus (flu)cloxacillin was calculated exclusively by using patients in this silo’s randomized comparison (Figure 1) with pre-specified information sharing from the 87 pediatric participants enrolled in this silo (study ongoing). The probability of non-inferiority (pre-specified as an aOR<1.2; approximating an absolute margin of 2.5% absolute difference if mortality in the [flu]cloxacillin arm was 15%) and superiority (aOR<1.0) were estimated. The primary analysis for the primary outcome excluded participants with missing 90-day mortality data (complete case analysis). Post hoc analyses included analyses where all participants whose day 90 mortality data was missing were assumed to have survived or to have died.

Binary secondary outcomes were analyzed similarly. We planned a time-to-event analysis using a Bayesian Weibull proportional hazards model; however, this model’s assumptions were not satisfied and so we present unadjusted Kaplan-Meier curves. Pre-specified subgroup analyses for the primary outcome were the presence or absence of endocarditis and the presence or absence of suspected/proven central nervous system (CNS) infection. During peer review, a post hoc subgroup analysis was conducted for a shorter and longer duration of pre-treatment (≤48 hours, 48-72 hours).

As analyses were conducted in a Bayesian framework, we report the means of the posterior distributions of treatment effects with 95% credible intervals and where appropriate associated posterior probabilities. No P-values were calculated, and no adjustment for multiple analyses was pre-specified. Therefore, for the secondary estimands, excluding safety outcomes, the 95% credible intervals should not be used to draw definitive conclusions.

#### Interim Analyses and Decision Rules

As a perpetual adaptive Bayesian platform, simulations were conducted to optimize trial operating characteristics such as number and timing of interim analyses, decision rule thresholds, and with a maximum ceiling sample size of 6,000 patients.^12^ We planned and conducted scheduled analyses after every 500 platform patients reached day 90 with pre-specified stopping rules at each analysis for each domain.^12^ For the MSSA silo of the backbone domain, if the posterior probability of non-inferiority for cefazolin was greater than 99% in adults, the independent data and safety monitoring committee (DSMC) could recommend stopping recruitment. If the posterior probability of superiority for adults was less than 1%, the DSMC could recommend stopping for futility. If no threshold was met, recruitment was to continue unless the DSMC had substantial safety concerns which were not prespecified. For the MSSA non-inferiority comparison, simulations indicated an approximate frequentist type I error of 2%.

On June 21, 2024, at the fourth planned interim analysis, the DSMC requested recruitment be paused in the MSSA silo because of a safety signal involving AKI. Subsequently, on August 5, 2024, they recommended closing the MSSA silo because the statistical trigger for non-inferiority had been met, and the safety signal persisted. On August 7, 2024, without additional knowledge on study outcomes, the trial steering committee accepted the recommendation.

#### Study Oversight

International coordination was provided by the University of Melbourne with the direction of a global trial steering committee.^11^ Within each participating region, study coordination and monitoring was provided by a sponsor who was responsible under each region’s laws and governance.

## Results

### Study Population

Of 8546 patients screened as of June 21, 2024, 2602 adults (30.4%) were recruited to the platform and underwent one or more randomizations (Fig. S1). Of those, 1757 had MSSA (67.5%) and 1341/1757 (76.3%) participants were randomized in the MSSA silo (671 to cefazolin, 670 to (flu)cloxacillin; Figure 1). Fifty-four of these patients (4.0%) were lost to follow-up, leaving 1287 patients in the primary outcome analysis. Recruitment to SNAP is ongoing and to maintain trial integrity, the results for the pediatric patients, MRSA silo, and other domains remain blinded.

Baseline characteristics are presented by treatment assignment in Table 1. The median age was 66 years (interquartile range [IQR] 53–76) and 421 (31.4%) were female, which are representative of a typical cohort with *S. aureus* bacteremia (Tables S1-S3). The most common site of infection was osteoarticular (32.1%) (Table 2). Antibiotics received prior to randomization allocation are presented in Table S4. Four hundred and eighty-eight patients (36.4%) were receiving cefazolin at enrollment and 665 (49.6%) (flu)cloxacillin, with similar numbers across the assigned treatment groups. More than 99% of patients in this study received infectious diseases consultations. Both treatment groups received a similar duration of intravenous and oral antibiotics (Table S5).

### Primary Outcome

Ninety-seven of 645 evaluable patients (15.0%) in the cefazolin group and 109 of 642 evaluable patients (17.0%) in the (flu)cloxacillin group met the primary outcome of mortality at 90 days (aOR 0.81; 95% CrI 0.59–1.12) corresponding to a posterior probability of non-inferiority of 99.2% and superiority of 89.8% (Table 3, Fig. S2). In the 3.8% of patients with missing primary outcome data, the baseline characteristics were similar to those with complete data, apart from a younger age and history of injecting drug use in both treatment arms (Table S6). The complementary analyses accounting for missing outcomes were congruent with the primary analysis having posterior probabilities of non-inferiority exceeding 99%. A further post hoc sensitivity analysis using a broader prior demonstrated similar results to the primary analysis (Table S7).

### Secondary Outcomes

Secondary outcomes are presented in Table 3. At every time point prior to day 90, cefazolin had a probability of non-inferiority for mortality exceeding 98.8% and a probability of superiority exceeding 94.1%. Unadjusted Kaplan-Meier plots for mortality are provided in Figure S3.

Patients receiving cefazolin experienced less AKI than those receiving (flu)cloxacillin (92/660 [13.9%] versus 127/648 [19.6%]; aOR 0.67; 95%CrI 0.50–0.89; probability of superiority 99.7%); including a 94.6% posterior probability of a reduced risk of initiating renal replacement therapy within 90 days (Table 3, Fig. S4). The staging of AKI is presented in the Table S9.

### Protocol-adherent and Prespecified Subgroup Analyses

The protocol-adherent analysis demonstrated a 95.4% probability of cefazolin being non-inferior to (flu)cloxacillin (Table 3). Mortality for patients with endocarditis was 8/51 (15.7%) for cefazolin and 13/53 (24.5%) for (flu)cloxacillin (aOR 0.54; 95%CrI 0.20–1.43; probability of non-inferiority 94.4% and superiority 88.9%). There were only 7 patients with a central nervous system infection, precluding meaningful comparison.

### Reported Adverse Events

Serious adverse reactions related to study drugs are detailed in Table 3 and Table S10. Overall, cefazolin had substantially lower odds of a serious adverse reaction being reported (aOR 0.41; 95%CrI 0.21–0.77) and of having to be discontinued due to adverse events (aOR 0.21; 95%CrI 0.11–0.38).

## Discussion

In this pragmatic randomized clinical trial in adults with MSSA bacteremia, cefazolin was non-inferior to (flu)cloxacillin for the primary outcome of all-cause mortality at 90 days. Cefazolin also had lower risk of acute kidney injury; initiation of renal replacement therapy; and of treatment discontinuation due to adverse events. These data also suggest that cefazolin may reduce mortality, with an estimated probability of superiority of 94–99% at days 14, 28, and 42 which remained approximately 90% by day 90.

The adjusted odds ratio for 90-day mortality of 0.81 (CrI 0.59–1.12) for cefazolin versus (flu)cloxacillin is similar to pooled estimates from observational studies of an overall odds ratio of 0.73 compared to the antistaphylococcal penicillins and 0.31 to 0.92 for individual drugs within the class.^9^ While patients with *S. aureus* bacteremia are at direct risk of acute kidney injury by virtue of acute illness,^17^ the potential nephrotoxicity of the antistaphylococcal penicillins has been recognized since the 1960s.^18^ While we used flucloxacillin and cloxacillin in this trial, other antistaphylococcal penicillins have similar antibacterial activity and chemical structures,^19^ and based on observational data are likely to confer a similar risk of nephrotoxicity or treatment discontinuation for adverse events when compared to cefazolin.^9^ Therefore, the mortality and nephrotoxicity findings reported here may apply to other antistaphylococcal penicillins other than (flu)cloxacillin.

Cefazolin has some potential disadvantages compared to (flu)cloxacillin, including higher drug acquisition costs in some markets, and a potentially broader antimicrobial spectrum. However, these concerns are balanced by its longer half-life, reduced toxicity, and possible superiority to (flu)cloxacillin.

### Limitations

For reasons of practicality and feasibility, this was an open-label trial; however, the primary outcome, all-cause mortality at 90 days, was objective and the decision criteria to stop the trial was based on pre-specified statistical rules. We had limited control over physician decisions post-randomization. In the presence of persistent bacteremia or other clinical factors, physicians could have done more diagnostic testing, changed or added antibiotics, or performed more aggressive source control. We did not collect and therefore cannot present these data. We donot believe post-randomization care would have been different by knowledge of treatment assignment, but this is a limitation of the open-label design and also that variability of practices across sites may have introduced heterogeneity. Fortunately, a change of assigned therapy due to clinician perceived treatment failure was less than 2.5% and the results of the protocol-adherent population analysis were consistent with the primary analysis. Second, we have not yet tested isolates for the presence of the cefazolin inoculum effect and will report this microbiological subgroup when testing of all isolates has been performed at central reference laboratories. In the interim, concerns about the clinical relevance of the cefazolin inoculum effect^7^ should be substantially allayed by the narrow non-inferiority margin and similar estimated treatment effect in patients with infective endocarditis compared to the overall population. Third, we presented a complete case analysis for the primary outcome (3.8% missing), with complementary analyses where all missing patients were considered either alive or dead yielding near identical results.

## Conclusion

This pragmatic international, open-label platform trial demonstrated that cefazolin was non-inferior to (flu)cloxacillin for 90-day all-cause mortality in the treatment of adults with MSSA bacteremia. Cefazolin was associated with fewer serious adverse reactions, in particular less acute kidney injury.

Disclosure forms provided by the authors are available with the full text of this article at NEJM.org.

## Supplementary Material

Supplement

## Figures and Tables

**Table 1:** Baseline Characteristics of Patients

	No./Total No.(%)	
Variable	Cefazolin (N=671)	(Flu)cloxacillin (N=670)
**Demographics**		
Median age (IQR)	66 (53−76)	66 (52−77)
Female sex	215 (32.0)	206 (30.7)
Median weight in kg (IQR)	82.0 (69.0−96.8)	80.0 (68.0−95.0)
**Comorbidities**		
Diabetes mellitus	231 (34.4)	217 (32.4)
Chronic kidney disease^1,2^	96 (13.9)	93 (14.3)
Cirrhosis	34 (5.1)	30 (4.5)
Cancer requiring any surgical or medical therapy within 12 months^3^	95 (14.2)	76 (11.3)
Dementia	23 (3.4)	32 (4.8)
Iatrogenic immunosuppression^4^	105 (15.6)	84 (12.5)
Implanted intravascular prosthesis or endovascular device^5^	64 (9.5)	58 (8.7)
Previous infective endocarditis	11 (1.6)	10 (1.5)
Underlying cardiac abnormality predisposing to endocarditis^6^	50 (7.5)	42 (6.3)
Injection drug use within 6 months	31 (4.6)	52 (7.7)
Limitations to care^7^	80 (11.9)	84 (12.5)
**Prognostic Factors**		
Median hours to index blood culture positivity (IQR)	15.7 (12.0−21.0)	15.3 (12.0−20.7)
Median hours from index blood culture to platform entry (IQR)	52.3 (43.5−65.5)	50.9 (42.3−64.7)
Median hours from index blood culture to silo entry (IQR)	62.2 (50.2−67.9)	61.1 (50.0−67.5)
Intensive care unit at time of recruitment	74 (11.0)	72 (10.7)
Median Pitt bacteremia score (IQR)	0 (0−1)	0 (0−1)
Median C-reactive protein (mg/L) (IQR)	192.0 (113.5−266.5)	203.1 (122−283.8)

1 Defined as estimated glomerular filtration rate below 50mL/min/1.72m^2;

2 Four patients (0.3%) were receiving baseline hemodialysis or peritoneal dialysis (protocol deviations). They were retained in the intention-to-treat population

3 Excluding non-melanoma skin cancers

4 Within past 3 months has received any of the following: prednisone >0.5mg/kg/day (or equivalent) for more than 14 days, cyclosporine, tacrolimus, or sirolimus, azathioprine, leflunomide, or mycophenolate, cyclosporine or methotrexate, monoclonal antibodies such as infliximab, cancer chemotherapy, bone marrow transplantation, other immunosuppressive agents equal to above, AIDS

5 Not including central lines whether temporary or tunneled

6 Excluding prosthesis above

7 Active orders or advance care directives limiting resuscitation and intensive care admission.

**Table 2:** Focus of Infection

	No./Total No.(%)	
Focus of Infection^1^	Cefazolin (N=671)	(Flu)cloxacillin (N=670)
Osteoarticular (including epidural abscess)	220 (32.8%)	210 (31.3%)
Septic arthritis and extra-axial osteomyelitis	167 (24.9%)	151 (22.5%)
Vertebral osteomyelitis and/or septic discitis	61 (9.1%)	59 (8.8%)
Epidural abscess	45 (6.7%)	49 (7.3%)
Orthopedic hardware-associated	34 (5.1%)	31 (4.6%)
Skin and soft tissue	177 (26.4%)	204 (30.4%)
Isolated skin and soft tissue	127 (18.9%)	136 (20.3%)
Intravascular catheter associated	103 (15.4%)	102 (15.2%)
Isolated intravascular catheter infection	91 (13.6%)	91 (13.6%)
Primary bacteremia (no other focus identified)	81 (12.1%)	80 (11.9%)
Endocarditis	55 (8.2%)	57 (8.5%)
Left-sided native valve	35 (5.2%)	33 (4.9%)
Right-sided native valve	13 (1.9%)	19 (2.8%)
Prosthetic valve	13 (1.9%)	9 (1.3%)
Pleuropulmonary	40 (6.0%)	42 (6.3%)
Other deep focus	27 (4.0%)	37 (5.5%)
Genitourinary	24 (3.6%)	22 (3.3%)
Native vascular or vascular graft	15 (2.2%)	16 (2.4%)
Unspecified	14 (2.1%)	13 (1.9%)
Cardiac or other implantable non-orthopedic device	12 (1.8%)	16 (2.4%)
Central Nervous System	5 (0.7%)	2 (0.3%)

1 Patients may have had more than one focus of infection except where isolated line and skin and soft tissue infection is specified

**Table 3:** Primary and Secondary Outcomes

	No./Total No.(%)				
Outcome or Analysis	Cefazolin (N=671)	(Flu)cloxacillin (N=670)	Median Adjusted Odds Ratio (95%Credible Interval)	Posterior Probability of Cefazolin Non-Inferiority	Posterior Probability of Cefazolin Superiority
**Primary Outcome - 90 Day Mortality**					
Primary Analysis Population	97/645 (15.0%)	109/642 (17.0%)	0.81 (0.59–1.12)	99.2%	89.8%
Assuming all missing were dead	123/671 (18.3%)	137/670 (20.4%)	0.83 (0.63–1.10)	99.6%	90.7%
Assuming all missing were alive	94/671 (14.5%)	109/670 (16.3%)	0.81 (0.59-1.11)	99.3%	90.5%
Protocol-adherent Population	81/589 (13.8%)	73/520 (14.0%)	0.88 (0.61–1.26)	95.4%	76.0%
**Secondary Outcomes**					
Acute Kidney Injury within 14 days^1^	92/660 (13.9%)	127/648 (19.6%)	0.67 (0.50–0.89)	>99.9%	99.7%
Hepatotoxicity within 14 days^2^	75/574 (13.1%)	78/562 (13.9%)	0.96 (0.68–1.35)	90.0%	59.7%
Any serious adverse reaction related to study drug	12/671 (1.8%)	33/670 (4.9%)	0.41 (0.21–0.77)	99.9%	99.8%
Day 14 Mortality	25/667 (3.7%)	37/665 (5.6%)	0.67 (0.39–1.11)	NR	NR
Day 28 Mortality	47/665 (7.1%)	70/665 (10.5%)	0.61 (0.41–0.91)	NR	NR
Day 42 Mortality	63/663 (9.5%)	86/662 (13.0%)	0.66 (0.46–0.94)	NR	NR
Change of antibiotic due to perceived inefficacy	14/671 (2.1%)	16/670 (2.4%)	1.05 (0.51–2.10)	NR	NR
Microbiologic Treatment Failure After Day 14	18/587 (3.1%)	12/566 (2.1%)	1.41 (0.71–2.78)	NR	NR
Diagnosis of New Foci of Infection after Day 14	28/623 (4.5%)	30/609 (4.9%)	0.87 (0.53–1.45)	NR	NR
Change of antibiotic due to adverse event	11/671 (1.6%)	61/670 (9.1%)	0.21 (0.11–0.38)	NR	NR
New Renal Replacement Therapy within 90 days	17/668 (2.5%)	27/657 (4.1%)	0.61 (0.33–1.11)	NR	NR
Ongoing Renal Replacement Therapy at 90 days	4/662 (0.6%)	3/643 (0.5%)	1.00 (0.31–3.16)	NR	NR
*C. difficile* infection^3^	14/664 (2.1%)	10/661 (1.5%)	1.28 (0.60–2.66)	NR	NR
Intravenous catheter complication requiring removal^4^	22/668 (3.3%)	35/665 (5.0%)	0.68 (0.39–1.16)	NR	NR

1 Defined as a creatinine increase ≥26.5 μmol/L (0.3mg/dL) or ≥50% from baseline

2 Defined as alanine transaminase or gamma-glutamyl transferase increasing above 2.5 times the upper limit of normal

3 Defined as having symptoms, a positive test, and receiving therapy

4 While on assigned therapy.

5 Posterior probabilities are not reported for secondary outcomes, with the exception of safety outcomes, and 95% credible intervals do not include adjustment for multiplicity.

## References

[1] Antimicrobial Resistance Collaborators (2023). Global mortality associated with 33 bacterial pathogens in 2019: a systematic analysis for the Global Burden of Disease Study 2019. Lancet.

[2] Bai A, Lo CKL, Komorowski A (2022). *Staphylococcus aureus* bacteremia mortality: A systematic review and meta-analysis. Clin Microbiol Infect.

[3] Holland TL, Raad I, Boucher HW (2018). Effect of Algorithm-Based Therapy vs Usual Care on Clinical Success and Serious Adverse Events in Patients with Staphylococcal Bacteremia: A Randomized Clinical Trial. JAMA.

[4] Westgeest AC, Buis DTP, Sigaloff KCE (2023). Global Differences in the Management of *Staphylococcus aureus* Bacteremia: No International Standard of Care. Clin Infect Dis.

[5] Baddour LM, Wilson WR, Bayer AS (2015). Infective Endocarditis in Adults: Diagnosis, Antimicrobial Therapy, and Management of Complications: A Scientific Statement for Healthcare Professionals From the American Heart Association. Circulation.

[6] Delgado V, Ajmone Marsan M, de Waha S (2023). 2023 ESC Guidelines for the management of endocarditis: Developed by the task force on the management of endocarditis of the European Society of Cardiology (ESC) Endorsed by the European Association for Cardio-Thoracic Surgery (EACTS) and the European Association of Nuclear Medicine (EANM). Eur Heart J.

[7] Miller WR, Seas C, Carvajal LP (2018). The Cefazolin Inoculum Effect Is Associated With Increased Mortality in Methicillin-Susceptible *Staphylococcus aureus* Bacteremia. Open Forum Infect Dis.

[8] Lee S, Kwon KT, Kim H-I (2014). Clinical implications of cefazolin inoculum effect and β-lactamase type on methicillin-susceptible *Staphylococcus aureus* bacteremia. Microb Drug Resist.

[9] Prosty C, Noutsios D, Lee TC (2025). Cefazolin versus Antistaphylococcal Penicillins for the Treatment of Methicillin-Susceptible *Staphylococcus aureus* Bacteremia: A Systematic Review and Meta-Analysis. Clin Microbiol Infect.

[10] Tleyjeh IM, Kashour T, Mandrekar J, Petitti DB (2021). Overlooked Shortcomings of Observational Studies of Interventions in Coronavirus Disease 2019: An Illustrated Review for the Clinician. Open Forum Infect Dis.

[11] Tong SYC, Mora J, Bowen AC (2022). The *Staphylococcus aureus* Network Adaptive Platform Trial protocol: New tools for an old foe. Clin Infect Dis.

[12] Mahar RK, McGlothlin A, Dymock M (2023). A blueprint for a multi-disease, multi-domain Bayesian adaptive platform trial incorporating adult and paediatric subgroups: the *Staphylococcus aureus* Network Adaptive Platform trial. Trials.

[13] Anpalagan K, Dotel R, MacFadden DR (2024). Does adjunctive clindamycin have a role in *Staphylococcus aureus* bacteremia? A protocol for the adjunctive treatment domain of the *S. aureus* Network Adaptive Platform (SNAP) randomized controlled trial. Clin Infect Dis.

[14] de Kretser D, Mora J, Bloomfield M (2024). Early oral antibiotic switch in *Staphylococcus aureus* bacteraemia: The *Staphylococcus aureus* Network Adaptive Platform (SNAP) Trial Early Oral Switch Protocol. Clin Infect Dis.

[15] Liu C, Bayer A, Cosgrove SE (2011). Clinical practice guidelines by the Infectious Diseases Society of America for the treatment of methicillin-resistant *Staphylococcus aureus* infections in adults and children: executive summary. Clin Infect Dis.

[16] Henderson A, Cheng MP, Chew KL (2023). A multi-site, international laboratory study to assess the performance of penicillin susceptibility testing of *Staphylococcus aureus*. J Antimicrob Chemother.

[17] Legg A, Meagher N, Johnson SA (2023). Risk Factors for Nephrotoxicity in Methicillin-Resistant *Staphylococcus aureus*. Bacteraemia: A Post Hoc Analysis of the CAMERA2 Trial. Clin Drug Investig.

[18] Baldwin DS, Levine BB, McCluskey RT, Gallo GR (1968). Renal Failure and Interstitial Nephritis Due to Penicillin and Methicillin. N Engl J Med.

[19] Klein JO, Finland M (1963). The New Penicillins. N Engl J Med.

