# Supplementary material for: Cefazolin versus Cloxacillin or Flucloxacillin for Methicillin-Susceptible Staphylococcus aureus bacteremia: A Randomized Clinical Trial
Source: N Engl J Med. Author manuscript; Available in PMC 2026 Jul 13. (PMC7619232; doi:10.1056/NEJMoa2506905)
Supplement: Supplement [file EMS213498-supplement-Supplement.pdf]

Contents

Collaborators **4**

SNAP Working Groups and Committees 4

Penicillin and Methicillin-Susceptible Silos Domain-Specific Working Group *4*

Global Trial Steering Committee *4*

Statistics Working Group *4*

Microbiology Working Group *4*

Paediatrics & Pregnancy Working Group *4*

People Who Inject Drugs Working Group *5*

Data and Safety Monitoring Committee *5*

Consumer Reference Groups / Patient Participation Groups *5*

Regional Trial Steering Committees *5*

Trial Management Groups *6*

Analytical Team *7*

SNAP Sites and Site Investigators and Support Staff *7*

Financial Support *12*

Author Contributions **13**

Acknowledgments **13**

Supplementary Methods **14**

Platform Design 14

Participant Flow: *15*

Platform Core & Backbone MSSA Silo Domain-Specific Eligibility Criteria 16

Platform Core Eligibility Criteria *16*

Backbone Domain MSSA Silo Eligibility Criteria *16*

Dosing for Cefazolin and (Flu)cloxacillin 18

Standard Recommended Dosing *18*

Dose Adjustments for Patients with Critical Illness *18*

Renally Adjusted Dosing *18*

Outcome Measures 20

Core Primary Outcome Measure *20*

Core Secondary Outcome Measures *20*

Backbone Domain MSSA Silo Secondary Outcome Measures *21*

MSSA Silo Microbiology Methods 23

Statistical Methods 25

Definitions of trial populations *25*

Definition of trial Treatment Policy Strategy population (intention to treat) for the primary outcome estimand as per Statistical Analysis Plan 25

Definition of protocol-adherent population as per Statistical Analysis Plan 25

Model fitting diagnostics for the hierarchical model: 26

Statistical Documents 27

Supplementary Results 28

Figures 28

Figure S1: Flowchart of all Platform Participants' progress through the platform 28

Figure S2: Posterior probability distribution of the treatment effect for cefazolin vs (flu)cloxacillin for 90-day all-cause mortality in the Treatment Policy Strategy population (intention to treat) 29

Figure S3: Kaplan-Meier Plot for All-Cause Mortality censored at Day 90 from platform entry. A. Y-axis from 0.0 to 1.0. B. Y-axis from 0.80 to 1.0. 30

Figure S4: Posterior probability distribution of the treatment effect for cefazolin vs (flu)cloxacillin for Acute Kidney Injury 32

Tables 33

Table S1: Representativeness of the Study Population 33

Table S2: Participants, by country, stratified by intervention 34

Table S3: Ethnicity of participants, by country, stratified by intervention 35

Table S4: List of antibiotics administered between index blood culture collection and platform entry 37

Table S5: Duration of clinician estimated number of antibiotic days at 90 days 39

Table S6: Baseline characteristics of participants with and without the primary outcome data, stratified by randomly allocated treatment group 40

Table S7: Sensitivity analysis of primary outcome using a broad prior. The primary analysis used priors for the intervention of N (0, 1^2) and the sensitivity analysis used priors for the intervention of N (0, 10^2) 41

Table S8: Post-hoc descriptive analysis of 90-day mortality in participants enrolled at 0-48 hours and 48-72 hours after index blood culture collection, stratified by intervention group 42

Table S9: Acute Kidney Injury (AKI) Stages stratified by intervention 43

Table S10: Serious Adverse Reactions stratified by intervention 44

Table S11: Protocol deviations stratified by intervention 47

SNAP Protocols, Statistical Appendix and Statistical Analysis Plan 49

SNAP Core Protocol Version 2.0 49

SNAP Domain Specific Appendix: Backbone Domain: Penicillin and Methicillin-Susceptible Silos Version 2.0 49

SNAP Statistical Analysis Appendix Version 2.0 49

SNAP Backbone Domain for PSSA and MSSA Silos Statistical Analysis Plan Version 1.2 ... 49

Collaborators

SNAP Working Groups and Committees

Penicillin and Methicillin-Susceptible Silos Domain-Specific Working Group

Todd C. Lee (Chair); Achim Kaasch; Andrew Henderson; Anna Goodman; Brendan McMullan; Genevieve McKew; Ilse Kouijzer; Miquel Ekkelenkamp; Nesrin Ghanem-Zoubi; Nynke de Jager; Joshua S. Davis; Steven Y. C. Tong

Global Trial Steering Committee

Zoe McQuilten (Independent Chair); Anna L. Goodman; Asha Bowen; Catherine Cosgrove; Cesar A Arias; Dafna Yahav; David Lye; David Paterson; Emily Gibson McDonald; Genevieve Walls; George Heriot; Hannah Carter; Hiroki Saito; Jaap ten Oever; Jason Roberts; Julie Marsh; Lynda Whiteway; Marc Bonten; Marjolein Hensgens; Matthew P. Cheng; Matthew Scarborough; Nick Daneman; James Owen Robinson; Roger J. Lewis; Sebastiaan van Hal; Steven Webb; Susan Morpeth; Todd C. Lee; Thomas Boyles; Steven Y. C. Tong; Joshua S. Davis

Statistics Working Group

Julie Marsh (Chair); Alistair McLean; Hannah Carter; Jonas Tverring; Rebecca Turner; Roger J. Lewis; Scott Berry; Steve Webb; Thomas Lumley; Todd C. Lee; Tom Snelling; David Price; Joshua S. Davis; Steven Y. C. Tong

Microbiology Working Group

Sebastiaan van Hal (Chair); Benjamin Howden; Dan Gregson; Eugene Athan; Geoff Coombs; Jennifer Grant; Ka Lip Chew; Matthew P. Cheng; Neil Stone; Owen Robinson; Stefano Giulieri; Susan Morpeth; Andrew Henderson; Yen-Ee Tan; Rispah Chomba; Diane Daniel; Joshua S. Davis; Steven Y. C. Tong

Paediatrics & Pregnancy Working Group

Asha Bowen (Chair); Amanda Gwee; Anita Campbell; Anna Goodman; Brendan McMullan; Clare Nourse; Emma Best; Erica Hardy; Isabelle Malhamé; Kevin Schwartz; Lesley Voss; Oded Scheuerman; Phil Britton; Rachel Webb; Srinivas Murthy; James Hatcher; Michael Meyer; Tobias Strunk; Jesse Papenburg; Brett Manley; Michael Stark; Jonathan Cohen; Alasdair Munro; Steven Y. C. Tong; Joshua S. Davis

People Who Inject Drugs Working Group

Andrew Stewardson (Co-chair); Gail Matthews (Co-chair); Adrian Dunlop; Anna Goodman; Archana Sud; Carly Botheras; Eugene Athan; Marianne Martinello; Martin Llewelyn; Rebecca Sutherland; Stephen Aston; Terence Wuerz; Steven Y C Tong; Joshua S Davis

Data and Safety Monitoring Committee

David Huang (Chair); Jason Connor; Thomas Holland; Joanne Langley; Erin McCreary

Consumer Reference Groups / Patient Participation Groups

The SNAP Trial has Consumer Reference Groups / Patient Participation Groups comprised of individuals with lived experience of *Staphylococcus aureus* bloodstream infection or sepsis, either as a patient or as a relative or friend of a patient. Key consumers have a leadership role, serving as chairs of the consumer groups and/or providing representation on the Global Trial Steering Committee (GTSC) and other working groups as required as a voting member, facilitating a two-way dialogue between the consumer groups and the GTSC, and ensuring consumer voices inform strategic trial-level decision-making.

Australia

Asha Bowen (Co-chair); Lynda Whiteway (Co-chair); Caitlin Alsop; Caitlin Symons; Jen Sandilands; Keerthi Anpalagan; Lauren Barina; Michelle O'Brien; Perry Cunningham; Rachel Watt; Joshua S Davis; Steven Y C Tong

United Kingdom

Jennifer Bostock (Chair); Kim Smith; Joanne Lloyd; John O’Brien; Philip Bell

Regional Trial Steering Committees

Australia

Andrew Henderson; Ben Rogers; Clare Nourse; Heather Wilson; Jane Davies; Joshua S. Davis Katie Flanagan; Matthew O'Sullivan; Nick Anagnostou; Owen Robinson; Steven Y. C. Tong

Canada

Matthew P Cheng; Peter Daley; Nick Daneman; Joshua S. Davis; Jennifer Grant; Dan Gregson; Jennie Johnstone; Todd C. Lee; Emily G. McDonald; Terence Wuerz

Europe

Achim Kaasch; Marc Bonten; Marjolein Hensgens; Janneke Verberk; Jonas Tverring

New Zealand

Colin McArthur; Diana McNeill; Genevieve Walls; Kate Grimwade; Lesley Voss; Max Bloomfield; Rachel Webb; Susan Morpeth; Viliame Tutone

Singapore

David Lye; I. Russel Lee; Ka Lip Chew; Partha Pratim De; Shirin Kalimuddin; Sophia Archuleta; Yen Ee Tan

United Kingdom

Anna L. Goodman; Anne Grette Martson; James Hatcher; Jennifer Bostock; Jonathan Underwood; Marta O Soares; Martin Llewelyn; Matthew Scarborough; Michael Marks; Neil Stone; Rebecca M Turner; Robert Tilley; Sarah Pett; Ewan Harrison; Steven Y. C. Tong

Trial Management Groups

Global

Lauren Barina; Jocelyn Mora; Susan Goulding; Asha Bowen; Caitlin Symons; Chiara Piazzese; Ethan Foo; Grace McPhee; Vi Nguyen; Grace Norton; Sean Ong; Joshua S. Davis; Steven Y. C. Tong

Australia

Lauren Barina; Jocelyn Mora; Susan Goulding; Christine Sommerville; Mia Di Virgilio; Asha Bowen; Caitlin Symons; Chiara Piazzese; Ethan Foo; Grace McPhee; Vi Nguyen; Grace Norton; Joshua S. Davis; Steven Y. C. Tong

Canada

Matthew P. Cheng; Nick Daneman; Jennie Johnstone; Todd C. Lee; Emily G. McDonald; Lina Petrella

Europe

Janneke Verberk, Jodie Ipenburg, Wing Kee Yiek, Kartikkumar Rathod, Rianne Jahja, Sebastiaan Hullegie

Israel

Dafna Yahav; Mical Paul; Neta Petersiel

New Zealand

Dana de Kretser; Genevieve Walls; Hannah Burden; Kate Wong She; Rachel Webb; Susan Morpeth;

Singapore

Russel Lee

South Africa

Tom Boyles

United Kingdom

Abinayah Baskaran; Dana De Kretser; Denise Ward; Fleur Hudson; Shabinah S Ali; Sophia M E Bradshaw

Analytical Team

Anna McGlothlin (Chair); Michael Dymock; Robert Mahar

SNAP Sites and Site Investigators and Support Staff

<u>**Australia**</u>

Alfred Hospital
James McMahon; Patricia Griffiths, Ranjit Samra; Mei Tang

Armadale Hospital
David We-Jen New

Austin Health
Natasha Holmes; Ash Thomas; Andrew Gador-Whyte; Satwik Motaganahalli; Fiona James; Claire Kaufman; Shayne Camilleri; Brennan Collis; Sam Thorburn; Anthony Longhitano; Christine Wade; Connie Lam; Jordan Kahn; Kate Drummond; Hannah Poole; Lani De Silva; Morgan Henri; Gabriel Paykin

Bendigo Heath
Andrew Alexander Mahony; Andrew Peter Gador-Whyte; Victoria G Hall; Raelene Vine; Judy Lamb; Cherie Ritchie; Kate Booth; Sloane Birrell; Linda Dreyer

Blacktown Hospital
Ravindra Dotel; Shobini Sivagnanam; Ian Wong; Kyunghee Park

Cairns Hospital
Simon Smith; Josh Hanson; Laura Prideaux; Tej Shukla; Sussan Conway

Concord Repatriation General Hospital
Genevieve McKew; Rohan Beresford; Elaine Cheong; Chin Yen Yeo; Tanya Sinha; Aidan McLachlan; Heeral Thakkar; Samuel Baumgart; Megan Ung

Eastern Health – Box Hill Hospital
Stephen D Guy; Daniel Forster; Beau Carr; Roy Chean; Sarah Sparham; Mehrab Hossain; Connie Lam; Joanne Peel

Fiona Stanley Hospital
Owen J Robinson; Laurens Manning; Fionnuala Murray; Henco Nel; Michelle England

Flinders Medical Centre
Nicholas Anagnostou; Catherine Malden; Linda Broadbent; Teddy Teo; Thomas McNeil; Kathleen Ly

Gold Coast Hospital Health Service
Peter Simos; Katherine Garnham; Kimberley Ulett; Kelly Baker; Kylie Alcorn; Magdalena Mausolf; Imogen Bellamy; John Gerrard; Lee Forman; Don Deshan Wijesinghe; Monique Hare; Jack Cross

Goulburn Valley Health
Thomas Schulz; Nilesh Bhilave; Mohammed Ashraful; Ainsley Robinson; Usman Tahir

Grampians Health
Robert J Commons; Raquel Cowan; Jeni Mitchell; Michael Tuohill; Alexander Zehnwirth; Elvina Ingrid

John Hunter Hospital

Joshua S Davis; Kellie Schneider; Khine Swe; Brian Chong; Melissa Owen; Robert George; Elizabeth Canale

Launceston General Hospital

Mohamad-Ali Trad; Jaimie Frazer; Harsimran Singh

Liverpool Hospital

Hong Foo; Vana Nagendra

Lyell McEwin Hospital

Mark Boyd; Rory Hannah; Oui Ju; Jason Teng; Teddy Teo

Monash Health – Medical Centre Campus

Ben Rogers; Jillian Lau; Jessica O'Bryan; Sandra Davies; Debbie Radi; Ian Woolley; Sushena Krishnaswamy; Christopher Robson; Stephanie Spring; Namraj Goire; Bryan Tan; Kate Lim; Mihiri Weerasuria; Shivani Bhatt; Ranjit Samara; Nupur Bajaj; Isaac Lee; Beth Morgan

Nepean Hospital

Archana Sud; Zoe Jennings; Vidthiya Menon; Michael Findlay

Redcliffe Hospital

Kevin O'Callaghan; Maree Duroux; Megan Ratcliffe; Samantha Shone; Anna Brischetto; Alexandra Melon; Kiernan May

Royal Brisbane and Women's Hospital

Bridget Barber; Daisy Lindsay; Mark Fahmy

Royal Darwin Hospital

Jane Davies; Alex Hinchcliff; Caitlin Kent; Matilda Clark; Jane Nelson; Rob Baird; Josh Francis; Rob Duguid; Ella Meumann; Holly Gardner; Catherine Marshall; Genevieve Martin; Nerida Moore; Emma Smith; Stephanie Naidu; Jennifer Yan; Joshua Morigi; Caitlin Burt; Bojana Simsic; Stuart Campbell

Royal Melbourne Hospital

Justin Denholm; Katherine Bond; Diana Velasquez Reyes; Neta Petersiel; Steven Tong

Royal Perth Hospital

Owen J. Robinson; Katherine Norton; Timothy J Whitmore

Royal Prince Alfred Hospital

Sebastiaan J van Hal; Blake Nield

St Vincent's Hospital Sydney

Gail Matthews; Zachary James Webb-Harvey; Greg Dore; Jeremy Brown; David Andresen; Scott Chapman; Debbie Marriott; Romeo Torres

Sunshine Coast University Hospital

David Sowden; Alexandra Melon; Jennifer Broom; Keat Choong; Shradha Subedi; Lawrence Huang; Adam Stewart; Thi Aung; Kathryn Wilks; Drew Farquhar

University Hospital Geelong

Eugene Athan; Carly Botheras; Daniel O'Brien; Cameron Jeremiah; Aaron Walton; Sarah Huffam; Callum Maggs; Nomvuyo Mothobi; Andrew Hughes; Caroline Bartolo

Western Health – Footscray Hospital & Sunshine Hospital

James Steven Molton; Benjamin John Smith; Adrian Robert Tramontana; Aaron Elijah Bloch; Harry N. Walker; Jack Skeggs; Karen Liu; Ian Satya Haryono

Westmead Hospital

Matthew Vincent Neil O'Sullivan; David Pham; Neela Joshi Rai; Ying Li; Shirisha Sriramoju; Helen Baxter; Charbel Wehbe; Hector Maxwell-Scott; Wiraaj Agnihotri; Alyssa Pradhan

Wollongong Public Hospital

Niladri Ghosh; Lennox Jeryzna; Sue Ellen Holmes

Canada

Eastern Regional Health Authority - Health Sciences Centre and St Clare's Mercy Hospital

Peter Daley

University of Calgary sites – Foothills Medical Centre, Peter Lougheed Centre, Rockyview Hospital, and South Health Campus

Ranjani Somayaji, Daniel B Gregson; Elissa Rennert-May, Renata Rehak; John M Conly

University of Manitoba sites – Health Sciences Centre Winnipeg, St. Boniface Hospital and Grace Hospital

Terence Wuerz; Sylvain A Lother; Gloria Vazquez-Grande Joel Nkosi; Barret Rush; Ryan Zarychanski; Philippe Lagacé-Wiens; Aditya Sharma

Hamilton Health Sciences – Hamilton General Hospital and Juravinski Hospital

Dominik Mertz; Leslie J Martin; Deborah L Yamamura; Nishma Singhal

Hôpital Régional de Saint Jérôme

Sébastien Poulin; Marilyse Drapeau; Véronique Lapointe; Jean-Daniel Talbot

Hôpital Fleurimont

Francois Lamontagne; Philippe Martin; Louis Valiquette; Alex Carignan

Jewish General Hospital

Leighanne O Parkes; Yves Longtin; Ling Yuan Kong

Kingston General Hospital

Anthony D Bai; Santiago Perez-Patrigeon; Evan W Wilson; Barbara Antuna-Puente

Michael Garron Hospital

Christopher Kandel; ; Jeff Powis; Maureen Taylor

McGill University Health Centre - Montreal General Hospital and Royal Victoria Hospital

Matthew P Cheng; Todd C Lee; Emily G McDonald; Isabelle Malhamé; Jesse Papenburg; Lina Petrella; Iman Huseen; Kristen Moran; Katryn Paquette

Mount Sinai Hospital

Jennie Johnstone; Michael Fralick

Niagara Health - St Catharines and Niagara Falls Site

Aidan R Findlater; Jennifer LY Tsang; Erick H Duan; David J McCullagh; Lisa L Patterson

Sunnybrook Health Sciences Centre

Nick Daneman; Robert A Kozak; Robert A Fowler

Surrey Memorial Hospital

Kevin Afra; Christopher Condin, Deveshi Deveshi; Nibi C Varghese

The Ottawa Hospital - Civic and General Campus

Derek R MacFadden; Leanne M Mortimer; Lauralyn McIntrye

Unity Health Toronto - St. Michael's Hospital

Matthew P Muller; Larissa M Matukas

Unity Health Toronto- St. Joseph's Health Centre

Kevin L Schwartz; Greg J German

University of Alberta Hospital

Stephanie W Smith; Karen Doucette; Wendy I Sligl

Vancouver General Hospital

Jennifer M Grant; David J Harris

Cité de la Santé Hospital

Stephanie Castonguay, Marco A Bergevin; Olivier Haeck, Mario Roussos, Esther Simoneau, Nathalie Rivest, Tuyen Nguyen

Richmond Hospital

Clement Kwok

Sault Area Hospital

Lucas Castellani

University Health Network - Toronto General Hospital and Toronto Western Hospital

Maria Kulikova; Noelle R Yee

University Hospital LHSC

Michael S Silverman

Europe

Jeroen Bosch Hospital

Thijs ten Doesschate; Eva Kolwijck; Marjan van Apeldoorn; Barbara Hagemeijer - van Tol; Amber van Oers-Hornix; Yannick Kaiser; Cas van der Made

Radboud UMC

Fleur Sinkeler; Roger Brüggemann; Marianne Hendriks; Lieke Preijers; Margot Polfliet

Rijnstate Hospital

Elisabeth H. Gisolf; Maurits van Meer

UMC Utrecht

Marjolein P.M. Hensgens; Berend van Welzen; Iris Koevoets

Israel

Beilinson Hospital

Noa Eliakim Raz; Vered Daitch; Maayan Huber-Karl; Genady Drozdinsky; Poran Itamar

Rambam Health Care Campus

Yael Dishon-Benattar; Mical Paul; Mordechai Grupper; Yoav Shtabholz; Judit Debora Olchowski; Yaakov Dickstein; Ami Neuberger; Anat Stern; Marina Raines; Mona Mustafa Hellou; Gilad Rozenberg; Maria Belopolskaya; Daniel Haber; Moran Szwarcwort; Dina Pollak; Sigal Mendelshon ; Naifeh Krayem ; Nesrin Ghanem-Zoubi

Sheba Medical Centre

Nadav Baharav; Anat Wieder-Finesod; Sharon Amit; Dana Yelin; Neta Shirin; Vladislav Litchevsky; Asaf Biber; Lior Nave; Miriam Raanan

New Zealand

Auckland City Hospital

Mark Hobbs; Elizabeth Briggs; Annabelle Donaldson; Simon Briggs; Tom Hills; Rebekah Lane; Mitzi Nisbet; Stephen Ritchie; Caroline O’Connor; Hiromi Nakamura; Kristian Misa; Archie Maclang

Middlemore Hospital - Counties Manukau

Christopher Luey; Tim Cutfield; Boris Yow; Rima Song; Michael Borrie; Andrew Taylor; Stephen McBride; Susan Taylor; Veronica Playle; Vivian Lai; Tina Mwembani; Katherine Given; Nikita Jain; Imogen Nolan; Hamish Wright; Ruth Bollen; Delanie Nepia; Wing Cheuk Chan; Viliame Tutone; Natasha Pool

Hutt Valley

Matthew Kelly; Melissa Tan; Andrea Peat

North Shore Hospital

Nick Gow; Narrinder Shergill; Nicola Davies; Dragana Drinkovic

Tauranga Hospital

Kate Grimwade; Euna Sahng; Kevin Chen; Jennifer Goodson; Joanna Gempton; Murray Robinson; Vivian Black; Michael Addidle; Diane Hanfelt-Goade; Vani Sathyendran

Waikato Hospital

Paul Huggan; Katherine Walland; Patrick Hugh McGann; Tracey Kunac; Graham Mills; Hani Mohd Adnan; Thomas Wong; Elliott Green; Camilla Howard; Mohammed Issa; Anneke Marais; Catherina L Chang

Wellington Hospital

Max Bloomfield; Chris Clews; James Chancellor; Rose Ann Yap; Joan Parama

<u>**Singapore**</u>

National University Hospital

Brenda Mae Alferez Salada; Sean J.W. Wu; Jolene Oon; Timothy Chia; Siok Ching Chia; Sophia Archuleta; Ka Lip Chew

Singapore General Hospital

Hei Man Wong; Thien Siew Yee; Jean Sim; Dorothy Ng; Candice Chan; Yvonne Chan; Au Zi Ning; Weng Chenxin; Christina Titin; Shirin Kalimuddin

Tan Tock Seng Hospital

David Lye; Po Ying Chia; Ray J.H. Lin; Wilnard Y.T. Tan; Stephanie Sutjipto; Dongdong Ren; Amandip S. Sahota; Barnaby E. Young; Jen Mee Long; Jonathan J. Lim; Shiau Hui Diong; I. Russel Lee; Partha Pratim De

<u>**South Africa**</u>

Helen Joseph Hospital

Tom Boyles; Rispah Chomba; Zanele Mkhabela; Jeremy Nel; Lauren Richards

<u>**United Kingdom**</u>

Cardiff and Vale University

Jonathan Underwood, Clemency J Nye; Bethan A Lloyd; Sharon A Frayling; Teriann Noah Evans; Jaydee DV Pangan; Jennifer Patterson; Nikolaos I Grigoratos; Michael T Boswell; Owen Seddon; Jessica Blackaby; Shanine Mitchell

Guys and St Thomas

Anna Goodman, Thomas Juniper; Alieza Rara; Tom Williams; Alejandro Perez; Kelley Seitter; Joseph Price; Adam Gray; Sufia Hossain; Hugh Kingston; Nishi Patel; Karen Bisnauthsing; Snehaa Anandan; Fatima Yaqub; Esther Babalola; Luong Hoang; Massimo Bonaiti; Lisa Bryan; John Klein

Hull University Teaching Hospitals

Nicholas Easom; Akaninyene Out; Alexander J Richards; Debra Smith; Gemma Walker; Donna Norton; Lorraine Cullen; Cameron Bowen; Patrick J Lillie; Delia M Bianco; Matthew Nottage; Phillipa Burns; Penelope Sellers; Gavin Barlow; Muhammad Musassir; Joe Suich; Chloe Walsh

Liverpool University Hospital

Stephen J Aston; Alastair Yeoh; Allayna Doherty; Danielle McLaughlan; Faustina Ravi; Amy Doyle; Mary Brodsky; Dorothy Culpa; Wafae Ouarch; Ang Li; Ian Quayle

North Bristol

Ed Moran; Ankur Gupta-Wright; Elena Bellavia; Jade King; Kelly Knight; Maisie Borrill; Zsolt Friedrich; Francis Mensah; Kirstie Bradburn; Teagan Barrett; Michael Wilson; Beverley Faulkney; Louise Solomon; Chloe Sun; Kate Helliker; Emma Dickason-Palmer; Dan Langley; Mahableshwar Albur; Edward Moseley

Oxford University Hospitals

Matthew Scarborough, Michelle Kumin; Musaiwale Kamfose; Mark Campbell

University College London Hospitals

Michael Marks, Neil Stone, Michelle Berkeley; Sarah Eisen; Florence Taylor; Rubin Rose-Key; Sally Amor; Lucy Wellings; Sarah Logan; Sarah Horne; Melissa Chowdhury; Anisha Mangtani; Charlotte Zheng; Gemma Peters

University Hospitals Bristol and Weston

Raje Dhillon; Fergus Hamilton

Financial Support

The SNAP trial has funding from several national health research funding bodies; the views expressed are those of the author(s) and not necessarily those of funders.

- The Australian National Health and Medical Research Council (NHMRC; Australia) – Grant numbers1184238, 2014900, and 2032628
- The Australian Medical Research Future Fund (MRFF; Australia) – Grant number 2017301
- The Canadian Institutes of Health Research (CIHR, Canada) – Grant numbers 433304, 466322, and 474605
- The Canadian Accelerating Clinical Trials Consortium (ACT AEC; Canada) – Grant number 467903
- The Health Research Council of New Zealand (HRC; New Zealand) – Grant number 20/344
- The Starship Foundation (New Zealand) – Grant number ASF2144_WEBB
- The National Institute for Health and Care Research (NIHR; United Kingdom) – Grant number 133719; the views expressed are those of the author(s) and not necessarily those of the UK NIHR or the Department of Health and Social Care.
- The Medical Research Council (MRC; United Kingdom) – Grant number MC_UU_00004/05
- The Singapore National Medical Research Council (NMRC; Singapore) – Grant number CTGIIT21nov-0002
- The National Health Institute (NIH; United States) – Grant number 1R01AI173138-01A1
- The Netherlands Organisation for Health Research and Development (ZonMw; Netherlands) – Grant number 10140022110014
- UMC Utrecht (Netherlands)

Author Contributions

Todd C. Lee, Joshua S. Davis and Steven Y.C. Tong wrote the paper with early input from all authors. Todd C. Lee wrote the first draft of the manuscript.

Robert Mahar, Grace Norton, Anna McGlothlin, and Michael Dymock analysed the data. Steven Y.C. Tong, Grace Norton, Robert Mahar, Anna McGlothlin, and Michael Dymock take responsibility for the integrity of the data and accuracy of the data analysis.

Concept and design: Penicillin and Methicillin-Susceptible Silos Domain Specific Working Group, Global Trial Steering Committee, and Statistics Working Group.

Data Collection: Site Investigators

Critical revision of the manuscript for important intellectual content: All authors.

Statistical analysis: Robert Mahar, Anna McGlothlin, and Michael Dymock

Administrative, technical, or material support: Global Trial Management Group and Regional Trial Management Groups

Acknowledgments

SNAP trial is an international platform with a large number of contributors.

Todd C. Lee, Joshua S. Davis and Steven YC Tong of the PSSA/MSSA Domain-Specific Working Group conceived of and wrote the protocol for this part of the SNAP trial and are thus joint lead senior authors for this article. Todd C. Lee wrote the first draft of the article and collated all further input and responses. Steven Y C Tong is the corresponding author.

All SNAP PSSA/MSSA Domain-Specific Working Group, Global Trial Steering Committee and Writing Committee members conceived of the idea and contributed to the design and final article preparation.

S. Y. C. Tong and J. S. Davis were instrumental in developing the original concept of the SNAP platform design and are senior SNAP platform leads.

Concepts, protocol design, and writing were improved, edited, and reviewed through the SNAP Global Trial Steering Committee (as composed at the time of drafting and writing) and authors are listed.

Staphylococcus aureus Network Adaptive Platform (SNAP) Study Group members listed may differ from the group listed on other manuscripts and are specific to this article. These are collaborating authors who contributed to the design of the protocol and funding applications in their regions though not directly to the writing of this article.

The authors would like to thank the Data Safety Monitoring Committee (DSMC) and the Consumer Reference Groups (CRGs) and Public Patient Involvement (PPI) Groups in all regions for their input into protocol design.

Supplementary Methods

Platform Design

The platform is made up of Domains and Silos:

- 'Domains' refer to grouped types of interventions which are being compared. Active domains during the time of platform recruitment for the current report were: backbone (the initial upfront intravenous antibiotic); Adjunctive Treatment; and Early Oral Switch.
- 'Silos' correspond to the different antimicrobial susceptibility profiles of the infecting *Staphylococcus aureus* isolate (penicillin-susceptible [PSSA], methicillin-susceptible, penicillin-resistant [MSSA], and methicillin-resistant [MRSA])

<table>
<tr><th rowspan="2">Silo</th><th colspan="3">Domain</th></tr>
<tr><th>Backbone Domain</th><th>Adjunctive Treatment Domain</th><th>Early Oral Switch Domain</th></tr>
<tr><td>PSSA</td><td>(Flu)cloxacillin*
vs
Benzylpenicillin</td><td rowspan="3">No Adjunctive Clindamycin*
vs
Adjunctive Clindamycin</td><td rowspan="3">Continued intravenous (IV) treatment*
vs
Early Oral Switch

At 7 days
At 14 days</td></tr>
<tr><td>MSSA</td><td>(Flu)cloxacillin*
vs
Cefazolin</td></tr>
<tr><td>MRSA</td><td>Vancomycin / Daptomycin*
vs
Vancomycin / Daptomycin plus Cefazolin</td></tr>
</table>

* indicates the 'control' intervention.

- Eligibility is assessed for the platform overall, and then specifically for each domain. Assessment for, and entry into, each domain can occur at different time points.
- See 'Participant Flow' below. Participants may participate in one or more domains.

Participant Flow:

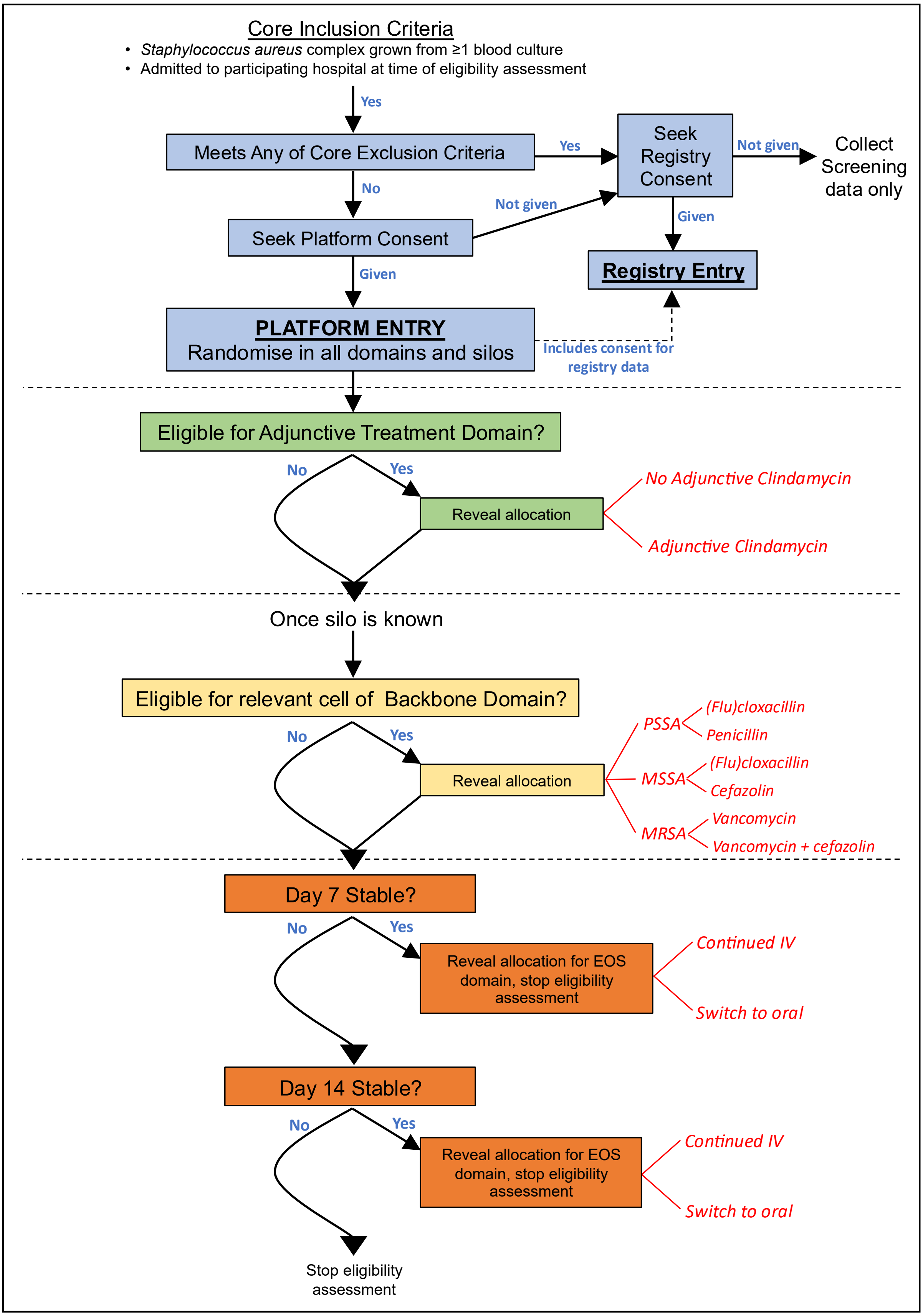

NB: Platform Entry = platform day 1; day of randomisation

Platform Core & Backbone MSSA Silo Domain-Specific Eligibility Criteria

Platform Core Eligibility Criteria

Inclusion criteria:

1. *Staphylococcus aureus* complex grown from ≥1 blood culture
2. Admitted to participating hospital at anticipated time of eligibility assessment (OR if patient has died, they were admitted to this site anytime from the time of blood culture collection until the time of eligibility assessment)

Exclusion criteria:

1. Time of anticipated platform entry is greater than 72 hours post collection of the index blood culture
2. Polymicrobial bacteraemia, defined as more than one organism (at species level) in the index blood cultures OR in any subsequent blood culture reported between the collection of the index blood culture and platform eligibility assessment, excluding those organisms judged to be contaminants by either the microbiology laboratory or treating clinician
3. Known previous participation in the randomised SNAP platform
4. Known positive blood culture for *S. aureus* (of the same silo: PSSA, MSSA or MRSA) between 72 hours and 180 days prior to the time of eligibility assessment
5. Treating team deems enrolment in the study is not in the best interest of the patient
6. Treating clinician believes that death is imminent and inevitable
7. Patient is for end-of-life care and antibiotic treatment is considered not appropriate
8. Patient <18 years of age and paediatric recruitment not approved at recruiting site
9. Patient has died since the collection of the index blood culture

Backbone Domain MSSA Silo Eligibility Criteria

Inclusion Criteria:

1. Index blood culture isolate is methicillin-susceptible but penicillin-resistant as per the Microbiology Appendix

Note that where trial sites are not testing for penicillin-susceptibility, patients with MSSA/PRSA on initial automated antimicrobial susceptibility testing (e.g., via Vitek2) can be included in the MSSA silo, but those with MSSA/PSSA (but not confirmed with a penicillin disc) will be excluded from the backbone domain. The rationale for this is that patients with MSSA but not tested with a penicillin disc may be truly PSSA (with no *blaZ*). If the cefazolin inoculum effect (CIE) is a clinically relevant entity, then including patients with an organism without *blaZ* (and hence cannot have a CIE phenotype), will bias towards non-inferiority of cefazolin compared to (flu)cloxacillin.

Exclusion Criteria:

1. >72 hours have elapsed from the time of index blood culture draw (i.e. the time of collection of the first positive blood culture from the patient during this episode)
2. History of type I hypersensitivity reaction (i.e. anaphylaxis or angioedema) to any penicillin or cephalosporin
3. History of severe delayed reaction (e.g. allergic interstitial nephritis, cutaneous vasculitis, Stevens-Johnson, Drug Reaction with Eosinophilia and Systemic Symptoms [DRESS], etc.) to any penicillin or cephalosporin
4. Non-severe rash to cefazolin or any penicillin (unless patient has been subsequently de-labelled)

   Nausea, diarrhoea, headache, and other non-specific symptoms are NOT allergies, they are drug intolerance, and they are not exclusion criteria. Similarly, a vague history of an allergy of unclear nature, or a family history of allergy are not exclusions.

5. Treating team deems enrolment in this domain is not in the best interest of the patient
6. Currently receiving maintenance dialysis (haemodialysis or peritoneal dialysis)

   Acute renal replacement therapy (including Continuous Renal Replacement Therapy [CRRT], haemodialysis or peritoneal dialysis) are not exclusions. Such patients are eligible as long as appropriate vascular access is available or can be arranged.

7. Polymicrobial bacteraemia (defined as more than one organism [at species level] in blood cultures, excluding those organisms judged to be contaminants by either the microbiology laboratory or treating clinician) reported between collection of the index blood culture and backbone domain eligibility assessment.
8. Patient currently being treated with a systemic antibacterial agent that cannot be ceased or substituted for interventions allocated within the platform (unless antibiotic is listed below, which specifies allowed antibiotics with limited absorption from the gastrointestinal tract or negligible antimicrobial activity against *S. aureus*)

   Continued use of the following antibacterial agents after the time of eligibility assessment will not constitute an exclusion criterion for enrolment in the trial. Topical administration of these or any other antibacterial agents is acceptable.

   - Antimycobacterial agents: Clofazimine, Dapsone, Capreomycin, Cycloserine, Ethambutol, Ethionamide, Isoniazid, Pyrazinamide
   - Miscellaneous agents: Colistin (enteral), Erythromycin (low dose erythromycin used as a pro-kinetic agent), Fidaxomicin, Fosfomycin (if dosed 3g once/week or less), Furazolidone, Neomycin (enteral), Nitrofurantoin, Paromomycin, Rifaximin, Tobramycin (enteral), Trimethoprim (≤300mg/d), Trimethoprim-sulfamethoxazole (prophylaxis i.e. ≤160mg/d TMP component average daily dose in adults or <4mg/kg/d in children), Vancomycin (enteral)

   This exclusion criterion was moved from core eligibility to backbone domain specific eligibility with protocol 1.0 to protocol 2.0 update.

Dosing for Cefazolin and (Flu)cloxacillin

Standard Recommended Dosing

Flucloxacillin:

Those randomized to (flu)cloxacillin, and flucloxacillin is available:

- (Flu)cloxacillin 2g every 6 hours intravenously

Cloxacillin:

Those randomized to (flu)cloxacillin, and cloxacillin but not flucloxacillin is available:

- Cloxacillin 2g every 4 hours intravenously

Cefazolin:

Those randomised to cefazolin

- Cefazolin 2g every 8 hours intravenously

Continuous infusion at 75-100% of the total daily dose was allowed as per local practice.

Dose Adjustments for Patients with Critical Illness

Critical illness defined as being admitted to ICU or having septic shock), endocarditis or central nervous system infection (includes brain or spinal cord infection, subdural empyema or CNS device-related infection, but does not include epidural abscess.

- (Flu)cloxacillin 2g every 4 hours intravenously

OR

- Cefazolin 2g every 6 hours intravenously

Renally Adjusted Dosing

Flucloxacillin:

GFR (mL/minute)	Standard Dose	High Dose (critical illness / infective endocarditis)
>50	2g q6h IV	2g q4h IV
10-50	2g q6h IV	2g q4h IV
<10	1g q6h IV	1g q6h IV

CRRT	2g q6h IV	2g q4h IV

NB: q6h refers to dosing every 6 hours

<u>Cloxacillin:</u>

There is no renal dosage adjustment necessary for any level of GFR or for CRRT.

<u>Cefazolin:</u>

**GFR (mL/minute)**	**Standard Dose**	**High Dose (critical illness/IE)**
>40	2g q8h IV	2g q6h IV
20-40	2g q12h IV	2g q12h IV
<20	1g q24h IV	1g q24h IV
CRRT	2g q12h IV	2g q12h IV

Outcome Measures

Core Primary Outcome Measure

The primary endpoint for all cells and domains will be all-cause mortality at 90 days after platform entry.

The primary endpoint will be determined through a search of hospital databases for a record of a participant's death, or follow-up contact with the participant's community healthcare provider, or follow-up contact with the patient or their nominated carer, or linkage with death registries.

Core Secondary Outcome Measures

The secondary outcomes in bold are reported in the current manuscript and as detailed in the 'SNAP Backbone Domain for PSSA and MSSA silos Statistical Analysis Plan Version 1.2' (see later in Supplemental Material). Other secondary outcomes will be reported in the future.

1. **All-cause mortality at 14, 28 and 42 days after platform entry**
2. **Duration of survival censored at 90 days after platform entry**
3. Length of stay of acute index inpatient hospitalisation for those surviving until discharge from acute inpatient facilities (excluding HITH/COPAT/OPAT/rehab), measured from platform entry to discharge from acute inpatient facilities, truncated at 90 days after platform entry
4. Length of stay of total index hospitalisation for those surviving until hospital discharge (including HITH/COPAT/OPAT/rehab), measured from platform entry to discharge from total index hospitalisation, truncated at 90 days after platform entry
5. Time to being discharged alive from the total index hospitalisation (including HITH/COPAT/OPAT/rehab), measured from platform entry to discharge from total index hospitalisation, truncated at 90 days after platform entry (and all deaths within 90 days will be considered '90 days')
6. **Microbiological treatment failure (Positive sterile site culture for *S. aureus* [of the same silo as the index isolate] between 14 and 90 days after platform entry)**
7. **Diagnosis of new foci between 14 and 90 days after platform entry. The presence of new foci will be determined by the site investigator and can incorporate clinical, radiological, microbiological and pathological findings.**
8. ***C. difficile* diarrhea as determined by a clinical laboratory in the 90 days following platform entry for participants ≥2 years of age**
9. **Serious adverse reactions in the 90 days following platform entry**
10. Health economic costs as detailed in the health economics appendix
11. Proportion of participants who have returned to their usual level of function at day 90 as determined by whether the modified functional bloodstream infection score (FBIS) remained the same or improved between baseline and 90 days after platform entry

12. Desirability of outcome ranking (DOOR) 1 (modified Antibiotic Resistance Leadership Group version) at 90 days after platform entry
13. Desirability of outcome ranking (DOOR) 2 (SNAP version) at 90 days after platform entry
14. **Total number of antibiotic days (IV and/or oral) in the 90 days following platform entry**
15. Days alive and free of antibiotics in the 90 days following platform entry

Backbone Domain MSSA Silo Secondary Outcome Measures

The secondary outcomes in bold are reported in the current manuscript and as detailed in the 'SNAP Backbone Domain for PSSA and MSSA Silos Statistical Analysis Plan Version 1.2' (see later in Supplemental Material). Other secondary outcomes will be reported in the future.

1. **Acute kidney injury defined using modified Kidney Disease Improving Global Outcomes (KDIGO) criteria.**

   The KDIGO guidelines for acute kidney injury (AKI) define AKI as:

   - Increase in serum creatinine by 0.3mg/dL (= 26.5 mmol/L) or more within 48 hours OR
   - Increase in serum creatinine to 1.5 times baseline or more within the last 7 days OR
   - Urine output less than 0.5 mL/kg/h for 6 hours

   For the purposes of SNAP, a modified KDIGO definition will be used:

   - Increase in serum creatinine by 0.3mg/dL (= 26.5 mmol/L) or more at any time from platform entry (baseline) to day 5 OR
   - Increase in serum creatinine by 1.5 times or more the level at platform entry (baseline) within 14 days of platform entry.

   Throughout the SNAP trial, day 1 is defined as the day of platform entry (i.e., the day the participant was deemed to be eligible, had consented, and was entered into the trial database).

   As a pragmatic trial, data collected on serum creatinine was mandated at platform entry (platform day 1 or the calendar day prior to platform entry) and days 5±1 and 14±3. Logistical complexities make it impossible to consistently collect data for urine output.

2. **Renal replacement therapy at any stage up to platform day 90.**
3. **Ongoing renal replacement therapy at platform day 90.**
4. **Hepatotoxicity - Grade 2 or above increase in ALT and/or GGT (>2.5x ULN), which was not present at platform entry, within 14 days after platform entry.**
5. **Change in assigned backbone antibiotic therapy during the total index hospitalisation, starting from reveal of allocated domain intervention, due to an adverse event deemed by the treating doctor/team to be of sufficient severity to change therapy.**
6. **Change in assigned backbone antibiotic therapy during the total index hospitalisation, starting from reveal of allocated domain intervention, due to presumed lack of efficacy according to the treating doctor/team**
7. **Peripherally inserted central catheter (PICC)/other central venous catheter complications requiring line removal, during the total index hospitalisation, starting from reveal of allocated domain intervention.**

This outcome will be collected at total index hospital discharge as a Y/N question. It will include any of the following: catheter-related blood stream infection; exit site infection; catheter-related superficial or deep venous thrombosis/thrombophlebitis; catheter blockage. It will NOT include PICC line rupture, leakage, displacement, or splitting unless it results in or occurs in addition to one of the above events.

Note that "total index hospitalisation" includes initial hospital admission to an acute inpatient facility, including HITH/OPAT and stepdown inpatient rehabilitation/post-acute care (if continuous with the initial inpatient admission).

MSSA Silo Microbiology Methods

Determine if the infecting *S. aureus* isolates is methicillin-resistant (MRSA), methicillin-susceptible, penicillin-resistant (MSSA/PRSA), or penicillin-susceptible (PSSA). From initial automated antimicrobial susceptibility testing (AST):

**Penicillin**	**Cefoxitin**	**Classification**	**Comments**
R	R	MRSA	No additional testing required
R	S	MSSA/PRSA	No additional testing required
S	S	PSSA	Penicillin disc test -> no beta-lactamase
		MSSA/PRSA	Penicillin disc test -> detects beta-lactamase

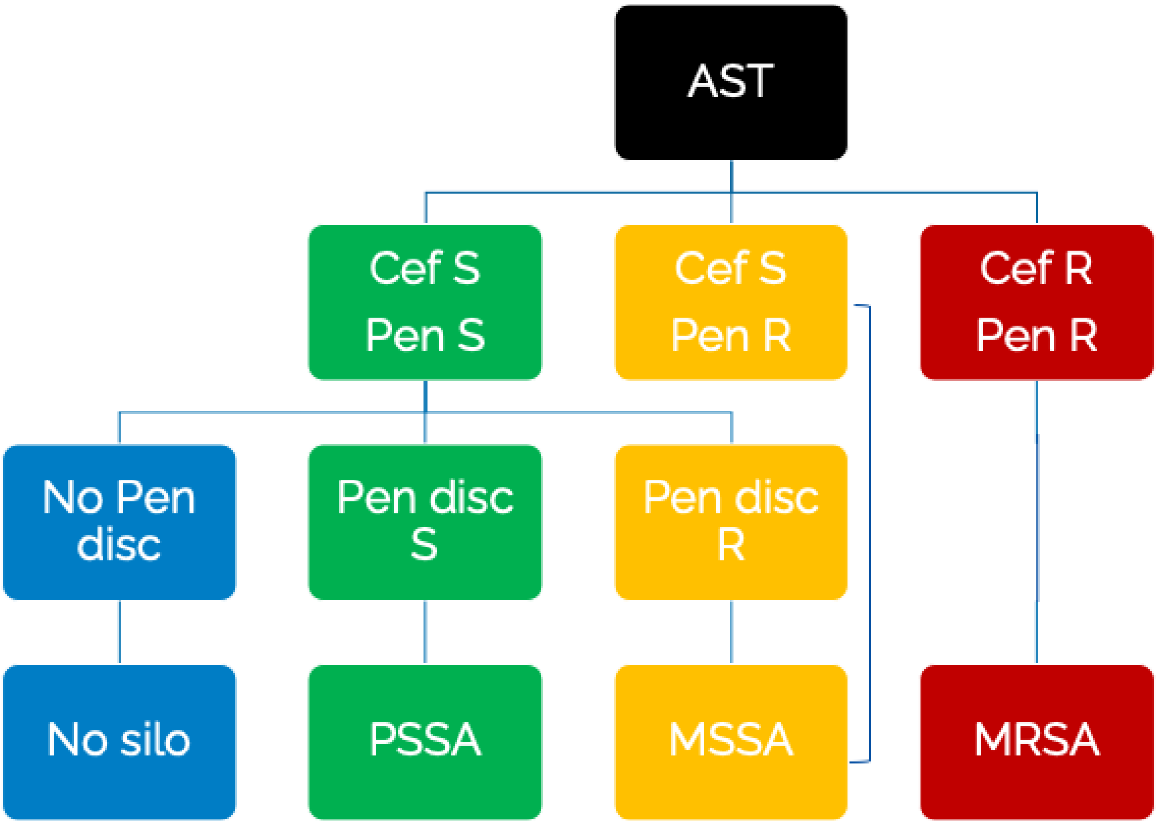

Where available, the use of PCR to detect the *mecA* gene could be used to identify MRSA.

34/91 sites used molecular methods (e.g., polymerase chain reaction [PCR]) for detection of the *mecA* gene to rapidly distinguish between MSSA and MRSA.

Patients can only be enrolled into the PSSA silo of the backbone domain if the laboratory performing susceptibility testing is able to perform confirmatory testing by either:

- Excluding a penicillinase producing isolate using either the EUCAST or CLSI penicillin disc (PD).
- Excluding the presence of the *blaZ* gene by PCR.

Please note that testing should be performed in a timely fashion to allow for enrolment within the 72-hr window and therefore it is suggested that the penicillin disc (PD) testing should be set up at the time of automated testing to allow for results to be available as soon as possible. Similarly, PCR workflows should be performed in real time.

For laboratories wishing to introduce testing, the Microbiology Working Group recommends introduction of the EUCAST method. A proposed workflow is outlined below. Although the EUCAST method is preferred over the CLSI method due to better sensitivity for *blaZ* detection, the CLSI method is an acceptable alternative.

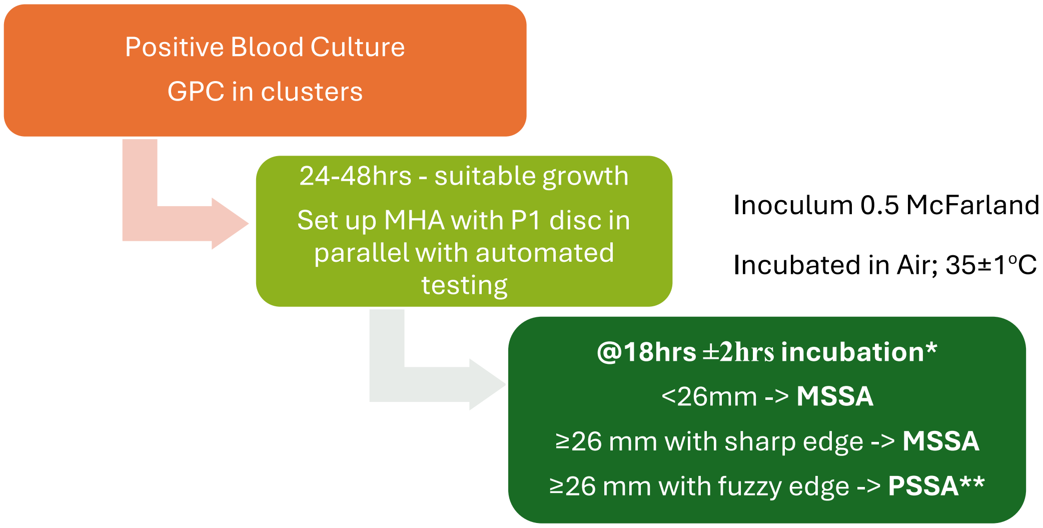

* See illustration below

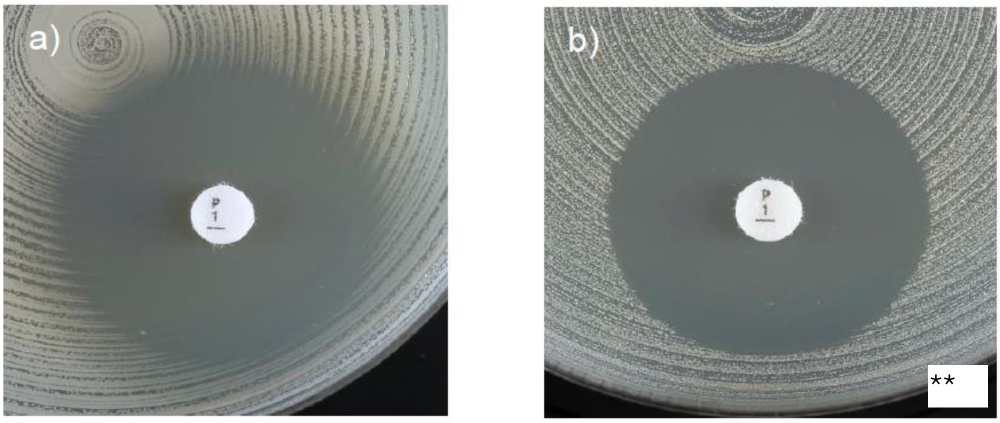

Examples of inhibition zones for *Staphylococcus aureus* with benzylpenicillin.
a) Fuzzy zone edge and zone diameter ≥ 26 mm. Report susceptible.
b) Sharp zone edge and zone diameter ≥ 26 mm. Report resistant.

**PSSA is confirmed if the zone diameter is ≥26 mm AND the zone edge is fuzzy.

In South Africa, a combination of a nitrocephin test with CLSI disc diffusion was used. If the nitrocephin test performed on day 1 growth detected the presence of penicillinase, the isolate was deemed to be penicillin-resistant, methicillin-susceptible. As the nitrocephin test is not sufficiently sensitive for detection of penicillinase, if the nitrocephin test did not detect the presence of penicillinase, then the CLSI disc diffusion method was used to determine the presence or absence of penicillinase.

Statistical Methods

Definitions of trial populations

Trial populations for the backbone domain are described separately for the PSSA and MSSA silos, which are mutually exclusive. The data for all platform analytic eligible participants are included in the analysis of each estimand, unless stated otherwise in the estimand sections. Overall eligibility for the backbone domain, for any silo, is included as a parameter in the primary statistical model.

Platform Analytic Eligibility	Participants who meet the inclusion and exclusion criteria detailed in the core protocol and who are classified as being members of one of the PSSA, MSSA or MRSA silos, based on the Microbiology at platform entry section below, and who have their randomisation revealed for at least one domain.
Backbone Domain Eligibility	Participants who are platform analytic eligible and meet the inclusion and exclusion criteria (including consent) for any of the backbone domain silos (PSSA, MSSA or MRSA).
MSSA (Silo) Eligibility	Platform eligible participants who meet the MSSA silo inclusion and exclusion criteria. Participants who are misclassified to the MSSA silo are only excluded from estimands based on target populations that adhere to protocol, eg. estimand A1.1.

Definition of trial Treatment Policy Strategy population (intention to treat) for the primary outcome estimand as per Statistical Analysis Plan

The Treatment Policy Strategy population is defined as those who met platform and domain eligibility criteria, excluding participants with missing mortality status at Day 90.

No adjustments will be made to the endpoint or the analysis as a result of permanently or temporarily stopping the intervention due to tolerance, efficacy, adherence or adverse events. Missing endpoint data may result from loss to follow-up or withdrawal or discharge to a subsequent acute care site where further data is unavailable. Participants with missing endpoint data are excluded from the analysis.

Definition of protocol-adherent population as per Statistical Analysis Plan

The protocol-adherent population is defined as those who met platform and domain eligibility criteria, without silo misclassification, and met the assigned intervention adherence criteria (below).

Silo misclassification could occur if the initial silo classification to PSSA, MSSA or MRSA at platform and domain entry was overturned with subsequent clinical laboratory work-up.

The adherence period for an individual is defined as the period from domain-specific randomisation reveal until the earliest of the following events:

- Platform day 14 (the last day that adherence is captured in the CRF)
- The date of allocation to early oral switch (this can occur on platform days 5-9 and 12-16)
- Date of death
- Date of discharge home
- Date of discharge to non-acute care
- Date of transfer to alternative acute care

All participants who do not meet the assigned intervention adherence criteria below will be defined as non-adherent, which includes participants who withdraw or are lost to follow-up prior to meeting any of these criteria.

Assigned to cefazolin:

Participants who adhere to treatment are defined as meeting the following conditions (in ranked order of evaluation):

- Adherence period is 6 days or fewer AND received at least the number of days in the adherence period minus one of (full or part) IV cefazolin AND received ≤1 day of IV (flu)cloxacillin
- Adherence period is 7 days or more AND received at least the number of days in the adherence period minus two of (full or part) IV cefazolin AND received ≤1 day of IV (flu)cloxacillin

Assigned to (flu)cloxacillin:

Participants who adhere to treatment are defined as meeting the following conditions (in ranked order of evaluation):

- Adherence period is 6 days or fewer AND received at least the number of days in the adherence
- period minus one of (full or part) IV (flu)cloxacillin AND received ≤1 day of IV cefazolin
- Adherence period is 7 days or more AND received at least the number of days in the adherence period minus two of (full or part) IV (flu)cloxacillin AND received ≤1 day of IV cefazolin

Model fitting diagnostics for the hierarchical model:

For the primary estimand, we performed posterior predictive checks of key marginal quantities (i.e. treatment, covariate, country, age, epoch effects etc.) and were satisfied with the fit graphically. When there were divergent transitions in the primary estimand model that were not resolved by standard methods, we confirmes that they were unlikely to influence model fit. Based on these diagnostics, for other binary outcomes we assumed that the model specification was appropriate. To maintain trial integrity, the diagnostic plots cannot be made public at this point in

time as their interpretation requires all model parameters to be reviewed, including parameters for interventions from domains that have not been closed or reported.

For all analyses, we confirmed adequacy of effective sample size of the computed posterior distribution and R-hats of all parameters. For the primary estimand model all R-hat values were < 1.01, 85% and 94% of model parameters had a normalised effective sample size of > 50% and > 10%, respectively. Where the normalised effective sample was less than 10%, mixing and convergence was confirmed by manual review of the MCMC traceplots. These diagnostics, taken together, indicate satisfactory convergence for the primary model.

For survival analyses we looked at typical log-log plots to evaluate whether the assumption of Weibull distribution was reasonable (it was not, in our opinion).

For ordinal analyses we assessed fit graphically using performed posterior predictive checks and Grotta plots.

Statistical Documents

All documents describing the statistical methods can be downloaded from the SNAP website: https://www.snaptrial.com.au/for-investigators#resource. The key documents are also provided in this Supplementary Appendix.

Supplementary Results

Figures

Figure S1: Flowchart of all Platform Participants' progress through the platform

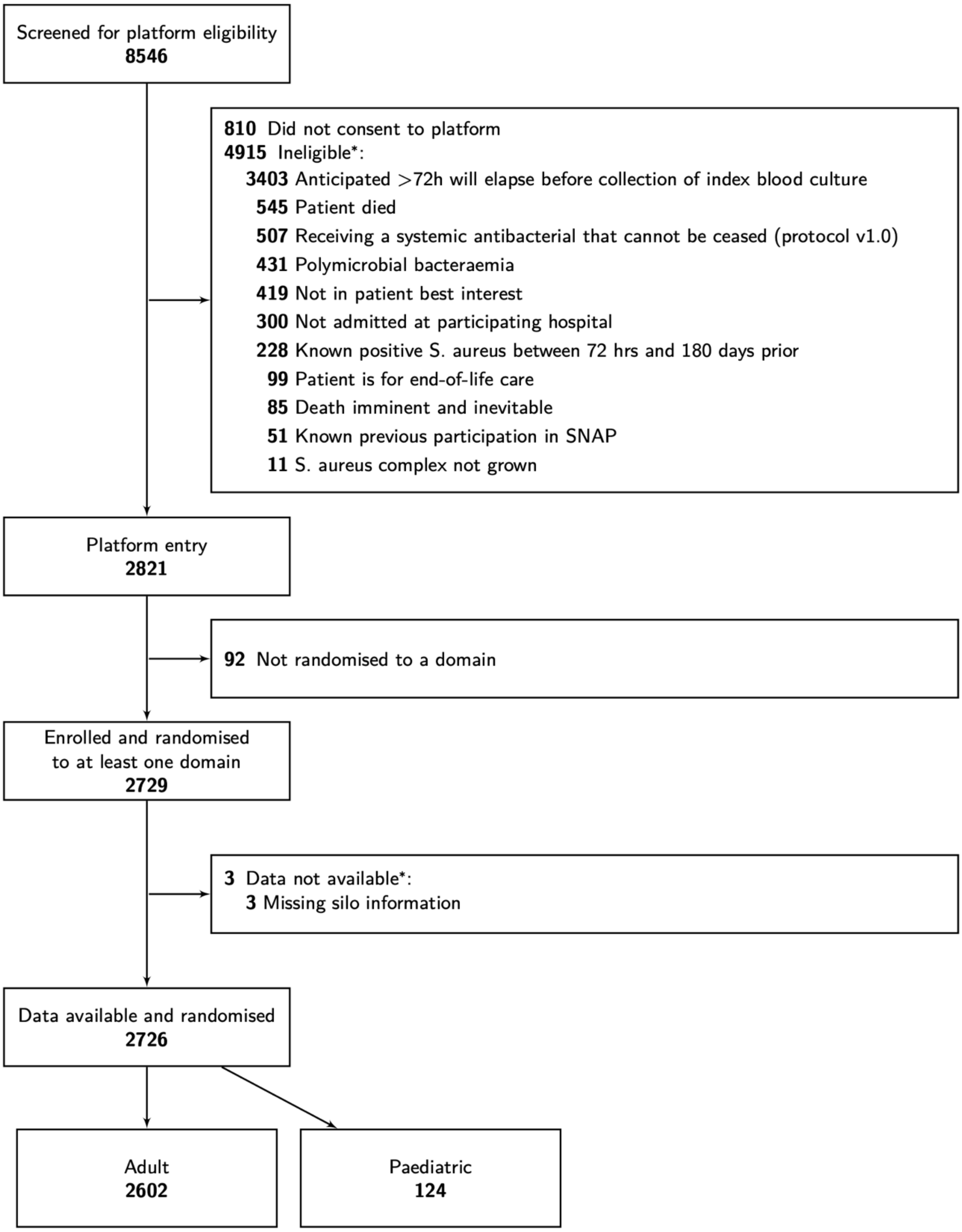

* Participants may meet more than one exclusion criteria.

Figure S2: Posterior probability distribution of the treatment effect for cefazolin vs (flu)cloxacillin for 90-day all-cause mortality in the Treatment Policy Strategy population (intention to treat)

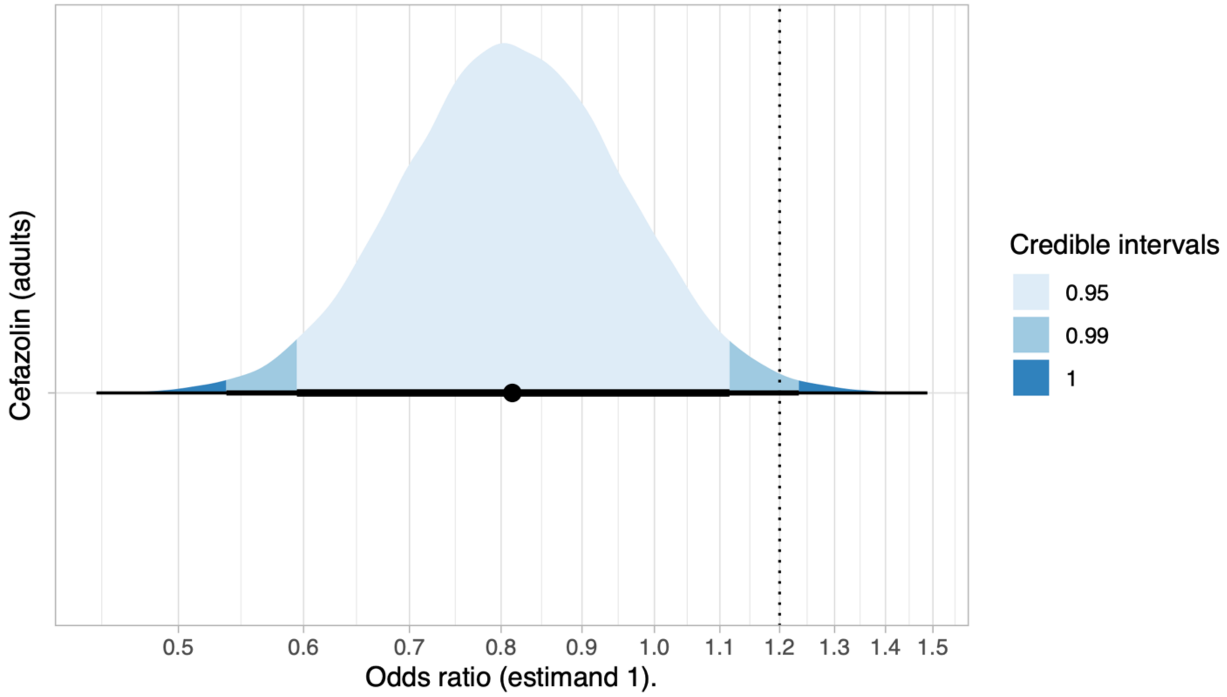

The dotted line refers to the pre-specified non-inferiority margin. The posterior probability of non-inferiority is the proportion of the distribution which lies to the left of the line at odds ratio of 1.2. The posterior probability of superiority is the proportion of the distribution curve which lies to the left of a line at odds ratio of 1.0.

Figure S3: Kaplan-Meier Plot for All-Cause Mortality censored at Day 90 from platform entry. A. Y-axis from 0.0 to 1.0. B. Y-axis from 0.80 to 1.0.

A.

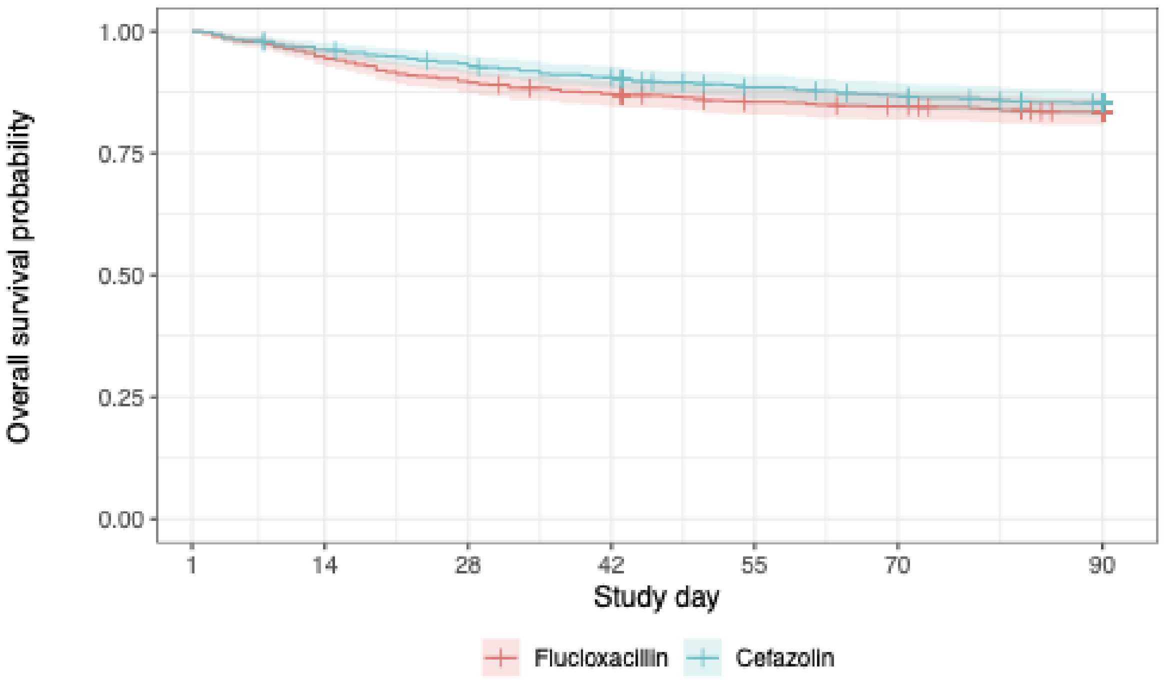

	1	14	28	42	55	70	90
**Flucloxacillin**							
At Risk	665	631	597	578	558	550	535
Events	0	37	70	86	96	102	109
Censored	0	0	0	2	11	13	556
**Cefazolin**							
At Risk	669	642	621	602	578	565	548
Events	0	25	47	63	76	88	97
Censored	0	2	4	6	15	17	572

B.

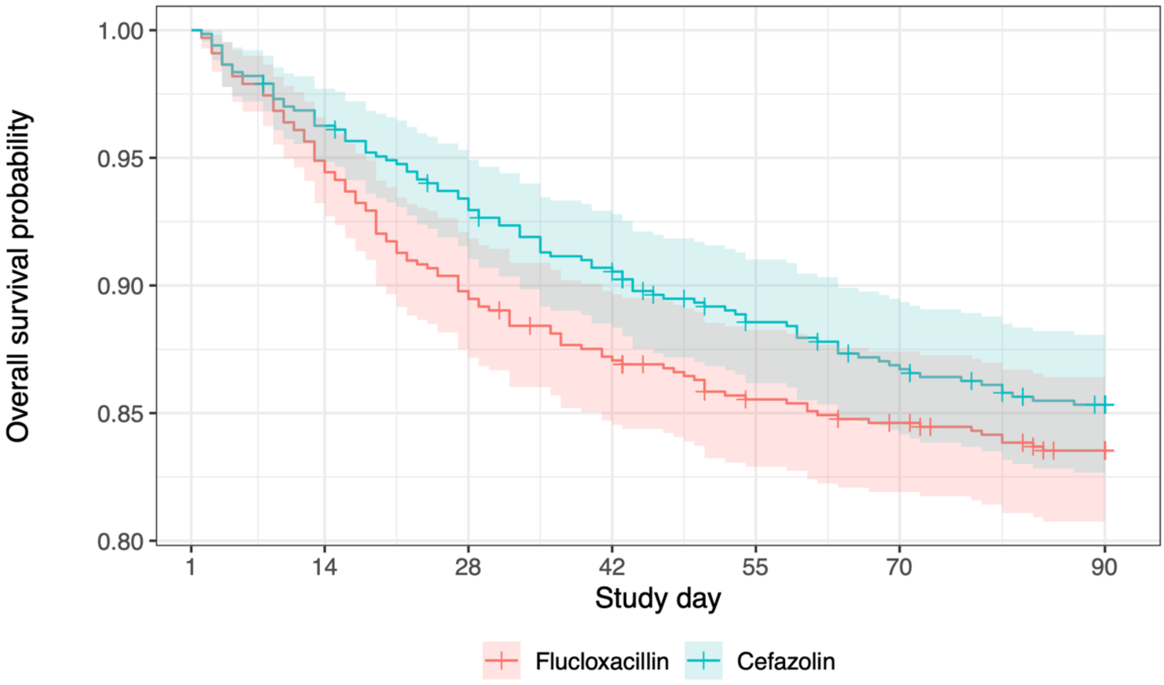

Flucloxacillin

At Risk	665	631	597	578	558	550	535
Events	0	37	70	86	96	102	109
Censored	0	0	0	2	11	13	556

Cefazolin

At Risk	669	642	621	602	578	565	548
Events	0	25	47	63	76	88	97
Censored	0	2	4	6	15	17	572

Figure S4: Posterior probability distribution of the treatment effect for cefazolin vs (flu)cloxacillin for Acute Kidney Injury

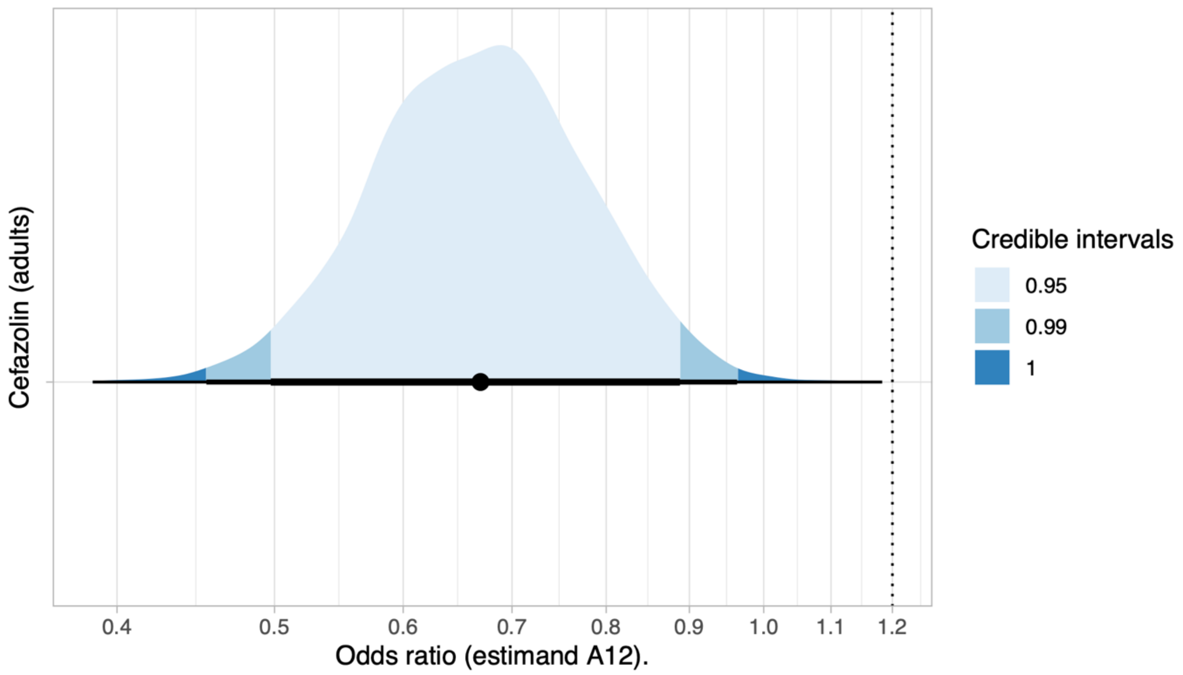

The posterior probability of non-inferiority is the proportion of the distribution which lies to the left of the line at odds ratio of 1.2. The posterior probability of superiority is the proportion of the distribution curve which lies to the left of a line at odds ratio of 1.0.

Tables

Table S1: Representativeness of the Study Population

**Condition under investigation**	*Staphylococcus aureus* bacteremia
**Special considerations related to:**	
Sex and gender	The proportion of participants who were female (421/1341, 31.4%) or male (920/1341, 68.6%) sex at birth were representative of previous reported cohorts of *S. aureus* bacteremia. The reason for this imbalance in incidence by sex at birth is not well understood.<br>Sex at birth was determined by participant self-report or from the medical records if self-report was not available. We did not collect data on gender.
Age	Participants were representative of reported cohorts of *S. aureus* bacteremia. The incidence of *S. aureus* bacteremia increases with age.
Geography	The trial was conducted in Australia, Canada, New Zealand, United Kingdom, Israel, Singapore, Netherlands, and South Africa. The majority of participants (n=1337) were from high income countries, with 4 participants from an upper-middle income country (South Africa).
Race or ethnic group	Data on ethnicity was collected specifically for each country, and as reported by participants or from the medical records if self-report was not available. These data are provided in Table S9 below.
People who inject drugs	Recent cohorts of *S. aureus* bacteremia comprise approximately 10% as people who inject drugs. 83/1341 (6.2%) of the trial participants reported injection drug use in the past 6 months. Thus, the people who inject drugs population were under-represented in this trial. Injection drug use was determined by participant self-report or from the medical records if self-report was not available.
Overall representativeness	The trial is an international trial enrolling participants from high and upper-middle-income countries. The representativeness by sex at birth and age are typical for *S. aureus* bacteremia. People who inject drugs are under-represented in the trial cohort.

Table S2: Participants, by country, stratified by intervention

Country, n (%)	Cefazolin (N=671)	(Flu)cloxacillin (N=670)
Australia	282 (42.0)	310 (46.3)
Canada	191 (28.5)	181 (27.0)
New Zealand	99 (14.8)	102 (15.2)
United Kingdom	40 (6.0)	30 (4.5)
Israel	36 (5.4)	19 (2.8)
Singapore	11 (1.6)	14 (2.1)
Netherlands	11 (1.6)	11 (1.6)
South Africa	1 (0.1)	3 (0.4)

Table S3: Ethnicity of participants, by country, stratified by intervention

Variable	Cefazolin (N=671)	(Flu)cloxacillin (N=670)
**Australia, n (%)**	**282 (42.0)**	**310 (46.3)**
Indigenous	13 (4.6)	11 (3.5)
Not Indigenous	264 (93.6)	297 (95.8)
Missing	5 (1.8)	2 (0.6)
**Canada, n (%)**	**191 (28.5)**	**181 (27.0)**
Black	4 (2.1)	6 (3.3)
Hispanic/Latino	0 (0.0)	3 (1.7)
Indigenous	4 (2.1)	5 (2.8)
Middle Eastern	2 (1.0)	3 (1.7)
South/South-East Asian	10 (5.2)	11 (6.1)
White	110 (57.6)	98 (54.1)
Unknown	53 (27.7)	48 (26.5)
Other	0 (0.0)	1 (0.6)
Missing	8 (4.2)	6 (3.3)
**New Zealand, n (%)**	**99 (14.8)**	**102 (15.2)**
Asian	3 (3.0)	3 (2.9)
Māori	18 (18.2)	23 (22.5)
Pacific Islander	17 (17.2)	14 (13.7)
White	52 (52.5)	52 (51.0)
Unknown	1 (1.0)	0 (0.0)
Missing	8 (8.1)	10 (9.8)
**United Kingdom, n (%)**	**40 (6.0)**	**30 (4.5)**
Asian	1 (2.5)	1 (3.3)
Black	2 (5.0)	0 (0.0)
White	36 (90.0)	28 (93.3)
Unknown	0 (0.0)	1 (3.3)
Other	1 (2.5)	0 (0.0)
Missing	0 (0.0)	0 (0.0)
**Israel, n (%)**	**36 (5.4)**	**19 (2.8)**

Jewish	27 (75.0)	17 (89.5)
Middle Eastern	8 (22.2)	2 (10.5)
Missing	1 (2.8)	0 (0.0)
**Singapore, n (%)**	**11 (1.6)**	**14 (2.1)**
Asian	7 (63.6)	11 (78.6)
South/South-East Asian	4 (36.4)	3 (21.4)
Missing	0 (0.0)	0 (0.0)
**Netherlands, n (%)**	**11 (1.6)**	**11 (1.6)**
Missing*	11 (100.0)	11 (100.0)
**South Africa, n (%)**	**1 (0.1)**	**3 (0.4)**
Missing	1 (100.0)	3 (100.0)

*Ethnicity is not collected in the Netherlands.

Table S4: List of antibiotics administered between index blood culture collection and platform entry

Variable	Cefazolin (N=671)	(Flu)cloxacillin (N=670)
No antibiotics received, n (%)	11 (1.6)	7 (1.0)
Missing, n (%)	1 (0.0)	2 (0.0)
**Antibiotics received, n (%)**	**N=659**	**N=661**
Amikacin	2 (0.3)	6 (0.9)
Amoxicillin	19 (2.8)	14 (2.1)
Amoxicillin/Clavulanate	58 (8.6)	66 (9.9)
Azithromycin	47 (7.0)	41 (6.1)
Cefadroxil	0 (0.0)	0 (0.0)
Cefazolin	249 (37.1)	239 (35.7)
Cefepime	4 (0.6)	5 (0.7)
Cefotaxime	1 (0.1)	1 (0.1)
Ceftazidime	3 (0.4)	0 (0.0)
Ceftazidime/Avibactam	1 (0.1)	0 (0.0)
Ceftolozane/Tazobactam	0 (0.0)	0 (0.0)
Ceftriaxone	184 (27.4)	196 (29.3)
Cefuroxime	18 (2.7)	15 (2.2)
Cephalexin	8 (1.2)	14 (2.1)
Ciprofloxacin	17 (2.5)	11 (1.6)
Clarithromycin	9 (1.3)	0 (0.0)
Clindamycin	23 (3.4)	24 (3.6)
Cloxacillin	13 (1.9)	15 (2.2)
Co-trimoxazole	6 (0.9)	3 (0.4)
Daptomycin	0 (0.0)	3 (0.4)
Dicloxacillin	2 (0.3)	0 (0.0)
Doxycycline	12 (1.8)	7 (1.0)
Ertapenem	3 (0.4)	1 (0.1)
Erythromycin	2 (0.3)	0 (0.0)
Flucloxacillin	323 (48.1)	314 (46.9)

Gentamicin	56 (8.3)	54 (8.1)
Imipenem	0 (0.0)	0 (0.0)
Lincomycin	1 (0.1)	0 (0.0)
Linezolid	3 (0.4)	0 (0.0)
Meropenem	13 (1.9)	20 (3.0)
Meropenem/Vaborbactam	0 (0.0)	0 (0.0)
Metronidazole	21 (3.1)	28 (4.2)
Moxifloxacin	0 (0.0)	0 (0.0)
Nitrofurantoin	0 (0.0)	0 (0.0)
Penicillin (Benzylpenicillin)	6 (0.9)	11 (1.6)
Piperacillin/Tazobactam	126 (18.8)	148 (22.1)
Rifampicin	0 (0.0)	1 (0.1)
Teicoplanin	2 (0.3)	3 (0.4)
Ticarcillin/Clavulanate	1 (0.1)	1 (0.1)
Vancomycin	303 (45.2)	303 (45.2)
Other	25 (3.7)	16 (2.4)

Table S5: Duration of clinician estimated number of antibiotic days at 90 days

	**Cefazolin (N = 645)**	**(Flu)cloxacillin (N = 642)**
Days of IV antibiotics (median, IQR)	26.0 (14.0, 42.0)	26.0 (14.0, 41.0)
Days of oral antibiotics (median, IQR)	7.0 (0.0, 28.0)	7.0 (0.0, 28.0)

Table S6: Baseline characteristics of participants with and without the primary outcome data, stratified by randomly allocated treatment group

Factor	Missing Primary Outcome - (Flu)cloxacillin Arm	Not Missing Primary Outcome - (Flu)cloxacillin Arm	Missing Primary Outcome - Cefazolin Arm	Not Missing Primary Outcome - Cefazolin Arm
Total, n	28	642	26	645
Female, n (%)	11 (39.3)	195 (30.4)	5 (19.2)	210 (32.6)
Age (median, IQR)	61.5 (41.8 - 75.3)	66.0 (53.0 - 77.0)	61.5 (44.3 - 68.8)	67.0 (54.0 - 76.0)
Focus of Infection - Infective Endocarditis, n (%)	4 (14.3)	53 (8.3)	4 (15.4)	51 (7.9)
Focus of Infection - Osteoarticular, n (%)	6 (21.4)	204 (31.8)	5 (19.2)	215 (33.3)
PWID - Yes, n (%)	7 (25.0)	45 (7.0)	7 (26.9)	24 (3.7)
Withdraw from future data collection, n (%)	5 (17.9)	0 (%)	5 (19.2)	0 (%)

Table S7: Sensitivity analysis of primary outcome using a broad prior. The primary analysis used priors for the intervention of N (0, 1^2) and the sensitivity analysis used priors for the intervention of N (0, 10^2)

	**Median adjusted OR**	**95% credible interval**
Primary analysis	0.81	0.59, 1.12
Sensitivity analysis	0.81	0.59, 1.12

Table S8: Post-hoc descriptive analysis of 90-day mortality in participants enrolled at 0-48 hours and 48-72 hours after index blood culture collection, stratified by intervention group

	**Cefazolin (N, %)**	**(Flu)cloxacillin (N, %)**
Enrolled 0-48h from index BC, with observed 90-day mortality outcome	255	273
Died by 90-days	44 (17.3)	49 (17.9)
Enrolled 48-72h from index BC, with observed 90-day mortality outcome	390	369
Died by 90-days	53 (13.6)	60 (16.3)

Table S9: Acute Kidney Injury (AKI) Stages stratified by intervention

Stage of AKI, n (%)	Cefazolin (N=671)	(Flu)cloxacillin (N=666)
No AKI	568 (84.6%)	521 (78.2%)
Stage 1	66 (9.8%)	89 (13.4%)
Stage 2	14 (2.1%)	19 (2.9%)
Stage 3	12 (1.8%)	19 (2.9%)
Missing data	11 (1.6%)	18 (2.7%)

The highest serum creatinine result was collected at three time points where available:

- baseline (platform day 1 or within 24 hours prior to platform entry)
- between platform day 1 and day 5 inclusive
- between platform day 6 and day 14 inclusive

AKI Stage 1 was defined as:

- Serum creatinine increase of 1.5 to <2.0 times from baseline to day 1–5, or baseline to day 6–14, or day 1–5 to day 6–14 OR
- Serum creatinine increase of ≥26.5μmol/L within the first 5 days (absolute difference between baseline and day 1–5) AND serum creatinine increase of <2.0 times between baseline to day 1–5

AKI Stage 2 was defined as:

- Serum creatinine increase of 2.0 to <3.0 times from baseline to day 1–5, or baseline to day 6–14, or day 1–5 to day 6–14

AKI Stage 3 was defined as:

- Serum creatinine increase of ≥3.0 times from baseline to day 1–5, or baseline to day 6–14, or day 1–5 to day 6–14

If AKI is detected in more than one time window, the most severe stage is applied to staging.

Table S10: Serious Adverse Reactions stratified by intervention

Serious adverse events anticipated to occur as a result of trial interventions were, in general, included as secondary safety endpoints (e.g., acute kidney injury). Additional serious adverse events (considered grade 3 or higher) were only recorded across all regions if they were attributable to one or more study interventions (i.e., serious adverse reactions [SARs]; attributable to a protocol-determined drug or strategy).

Event type is summarised by medDRA Preferred Term (PT).

Variable	Cefazolin (N=671)	(Flu)cloxacillin (N=670)
Events (n)	14	37
**Days to onset (from platform entry)**		
Median (IQR)	12.0 (6.2, 16.8)	6.0 (3.0, 15.0)
Missing	0 (0.0)	0 (0.0)
**Event type, n (%)[a]**	**N=671**	**N=670**
Acute kidney injury	2 (0.3)	12 (1.8)
Administration site extravasation	0 (0.0)	1 (0.1)
Cardiac failure	0 (0.0)	1 (0.1)
Cholestatic liver injury	0 (0.0)	1 (0.1)
Clostridium difficile colitis	2 (0.3)	1 (0.1)
Cutaneous vasculitis	2 (0.3)*	0 (0.0)
Cytopenia	0 (0.0)	0 (0.0)
Delirium	0 (0.0)	0 (0.0)
Device related bacteraemia	0 (0.0)	0 (0.0)
Diarrhoea	2 (0.3)	0 (0.0)
Drug eruption	1 (0.1)	7 (1.0)
Drug hypersensitivity	0 (0.0)	0 (0.0)
Drug reaction with eosinophilia and systemic symptoms	0 (0.0)	0 (0.0)
Drug-induced liver injury	1 (0.1)	4 (0.6)
Extravasation	0 (0.0)	0 (0.0)
Headache	0 (0.0)	0 (0.0)
Hepatic enzyme increased	0 (0.0)	1 (0.1)
Hepatic failure	0 (0.0)	0 (0.0)

Hepatotoxicity	1 (0.1)	1 (0.1)
Hyperkalaemia	0 (0.0)	0 (0.0)
Hypervolaemia	0 (0.0)	0 (0.0)
Hypokalaemia	0 (0.0)	2 (0.3)
Hyponatraemia	0 (0.0)	0 (0.0)
Lung neoplasm	0 (0.0)	0 (0.0)
Metabolic acidosis	0 (0.0)	1 (0.1)*
Neutropenia	0 (0.0)	1 (0.1)
Pyrexia	0 (0.0)	0 (0.0)
Pyroglutamic acidosis	0 (0.0)	1 (0.1)
Rash maculo-papular	0 (0.0)	0 (0.0)
Somnolence	0 (0.0)	0 (0.0)
Suspected drug-induced liver injury	0 (0.0)	0 (0.0)
Thrombophlebitis	0 (0.0)	0 (0.0)
Tubulointerstitial nephritis	1 (0.1)	2 (0.3)
Vasculitic rash	0 (0.0)	1 (0.1)
Vomiting	2 (0.3)	0 (0.0)
Missing	0 (0.0)	0 (0.0)
**Grade, n (%)[b]**	**N=14**	**N=37**
3	13 (92.9)	35 (94.6)
4	1 (7.1)	1 (2.7)
5	0 (0.0)	1 (2.7)
Missing	0 (0.0)	0 (0.0)
**Outcome, n (%)[b]**	**N=14**	**N=37**
Ongoing	1 (7.1)	7 (18.9)
Recovered/resolved	8 (57.1)	25 (67.6)
Recovered with Sequelae	2 (14.3)	1 (2.7)
Death	3 (21.4)	2 (5.4)
Missing	0 (0.0)	2 (5.4)
**Association with Treatment, n (%)[b]**	**N=14**	**N=37**
Definitely	0 (0.0)	11 (29.7)

Probably	3 (21.4)	15 (40.5)
Possibly	11 (78.6)	11 (29.7)
Missing	0 (0.0)	0 (0.0)
**Suspected Unexpected Serious Adverse Reaction (SUSAR), n (%)**	**N=14**	**N=37**
Yes	1 (7.1)	1 (2.7)
Missing	0 (0.0)	0 (0.0)
**Significant Safety Issue (SSI), n (%)**	**N=14**	**N=37**
Yes	0 (0.0)	0 (0.0)
Missing	0 (0.0)	0 (0.0)

NB: Day of event from platform entry

NB: A single participant may have more than one SAR event reported

a. Proportions with total participants as the denominator (excluding missing data)

b. Proportions with total number of serious adverse reactions as the denominator

* Event considered a SUSAR. There was one case of cutaneous vasculitis associated with cefazolin which was considered a SUSAR and one case of metabolic acidosis associated with (flu)cloxacillin that was considered a SUSAR.

Table S11: Protocol deviations stratified by intervention

The SNAP trial protocols allow for clinical decisions to be respected regarding patient management. In general, if the change in patient management is due to a clinical decision, this does not need to be reported as a protocol deviation, and the change in management will be captured in the Case Report Forms (CRFs).

For this trial, a protocol deviation has occurred when an event deviates from the study protocol AND one or more of the following three conditions are met:

1. The deviation is due to an error or mistake, OR
2. The deviation is due to treating clinician override, OR
3. The deviation is a trial-specified protocol deviation, namely:
    a. Day 2 (+/- 1) blood culture not completed – for adult participants only
    b. Day 90 vital status not entered into the database by platform Day 100
    c. Domain eligibility not assessed within the allocated time window(s)

Protocol deviations are reported for this manuscript if the deviation related to the core platform or to the backbone domain.

**Variable**	**Cefazolin (N=671)**	**(Flu)cloxacillin (N=670)**
Deviations, n(%)[a]	82 (12.2)	77 (11.5)
**Day of deviation (median, IQR)**		
Median (IQR)	**6.5 (2.0, 92.8)**	**4.0 (1.0, 100.0)**
Missing	0 (0.0)	0 (0.0)
**Type of Deviation, n (%)[a]**	**N=671**	**N=670**
Incorrect administration (type, duration, route), cessation, or non-administration of study drug	13 (1.9)	17 (2.5)
Day 2 (+/-1) blood cultures not done	8 (1.2)	7 (1.0)
Randomisation (into the core platform or a domain) of ineligible participant	9 (1.3)	11 (1.6)
Domain eligibility not assessed within the allocated time window(s)	0 (0.0)	0 (0.0)
Day 90 vital status not entered into the database by platform day 100	32 (4.8)	34 (5.1)
Other	20 (3.0)	8 (1.2)
Missing	0 (0.0)	0 (0.0)
**Impacts data[c], n (%)[b]**	**N=82**	**N=77**
Yes	21 (25.6)	12 (15.6)
Missing	0 (0.0)	0 (0.0)

**Impacts safety[d], n (%)[b]**	**N=82**	**N=77**
Yes	5 (6.1)	2 (2.6)
Missing	0 (0.0)	0 (0.0)
**Serious breach[e], n (%)[b]**	**N=82**	**N=77**
Yes	6 (7.3)	1 (1.3)
Missing	0 (0.0)	0 (0.0)

NB: Day of deviation is the day of event from platform entry

NB: A single participant may have more than one protocol deviation event reported

a. Proportions with total participants as the denominator (excluding missing data)

b. Proportions with total number of deviations as the denominator

c. The principal site investigator determined that the protocol deviation could potentially impact the completeness, accuracy and/or reliability of key (or critical) protocol identified data or processes

d. The principal site investigator determined that the protocol deviation could potentially impact a participant's rights, safety, or well-being.

e. A serious breach is any deviation of the approved protocol version or the clinical trial regulation that is likely to affect the rights, safety, or well-being of the trial participant and/or data reliability and robustness to a significant degree.

SNAP Protocols, Statistical Appendix and Statistical Analysis Plan

SNAP Core Protocol Version 2.0

SNAP Domain Specific Appendix: Backbone Domain: Penicillin and Methicillin-Susceptible Silos Version 2.0

SNAP Statistical Analysis Appendix Version 2.0

SNAP Backbone Domain for PSSA and MSSA Silos Statistical Analysis Plan Version 1.2
